# Inhibition of FAM19A5 reverses synaptic loss and cognitive decline in mouse models of Alzheimer’s disease

**DOI:** 10.1186/s13195-025-01813-8

**Published:** 2025-07-21

**Authors:** Han-Byul Kim, Sangjin Yoo, Hoyun Kwak, Shi-Xun Ma, Ryunhee Kim, Minhyeok Lee, Nui Ha, Soonil Pyo, Soon-Gu Kwon, Eun-Ho Cho, Sang-Myeong Lee, Juwon Jang, Won Kyum Kim, Hae-Chul Park, Minkyung Baek, Yosub Park, Ji-Young Park, Jin-Woo Park, Sun Wook Hwang, Jong-Ik Hwang, Jae Young Seong

**Affiliations:** 1 Neuracle Science Co., Ltd, Seoul, 02841 Republic of Korea; 2 Department of Biological Sciences, Korea Advanced Institute of Science and Technology, 291 Daehak-ro, Yuseong-gu, Daejeon, 34141 Republic of Korea; 3 Department of Biomedical Sciences, Graduate School of Medicine, Korea University, Ansan, 425-707 Gyeonggi-do Republic of Korea; 4 School of Biological Sciences, Seoul National University, Seoul, 08826 Republic of Korea; 5 Department of Clinical Pharmacology and Toxicology, Anam Hospital, Korea University College of Medicine, Seoul, 02841 Republic of Korea; 6 Department of Neurology, Korea University Anam Hospital, Korea University Medicine, Seoul, 02841 Republic of Korea; 7 Division of Clinical Pharmacology, Department of Medicine, Vanderbilt University Medical Center, Nashville, TN 37232 USA; 8 Department of Biomedical Sciences, Graduate School of Medicine, Korea University, Seoul, 02841 Republic of Korea

**Keywords:** FAM19A5, LRRC4B, NS101, Alzheimer’s disease, Mushroom spine, Synapse restoration

## Abstract

**Background:**

FAM19A5 is a secretory protein primarily expressed in neurons. Although its role in synaptic function has been suggested, the precise molecular mechanisms underlying its effects at the synapse remain unclear. Given that synaptic loss is a critical hallmark of Alzheimer’s disease (AD), elucidating the mechanisms involving FAM19A5 could provide valuable insights into reversing synaptic loss in AD.

**Methods:**

The binding partner of FAM19A5 was identified through co-immunoprecipitation experiments of mouse brain tissue. The effect of FAM19A5 on spine density in hippocampal neurons was evaluated using immunocytochemistry by overexpressing FAM19A5, treating neurons with FAM19A5 protein, and/or an anti-FAM19A5 antibody NS101. Target engagement of NS101 was determined by measuring FAM19A5 levels in mouse, rat, and human plasma at specific time points post NS101 injection using ELISA. Changes in spine density and dynamics in P301S tauopathy mice were assessed via Golgi staining and two-photon microscopy after NS101 administration. The synaptic strengthening of hippocampal neurons in APP/PS1 amyloidopathy mice after NS101 treatment was assessed by measuring miniature excitatory postsynaptic currents (mEPSCs) and field excitatory postsynaptic potentials (fEPSPs). Cognitive performance in AD mice after NS101 treatment was measured using the Y-maze and Morris water maze tests.

**Results:**

FAM19A5 binds to LRRC4B, a postsynaptic adhesion molecule, leading to reductions in spine density in mouse hippocampal neurons. Inhibiting FAM19A5 function with NS101 increased spine density. Intravenous administration of NS101 increased spine density in the prefrontal cortex of P301S mice, which initially showed reduced spine density compared to wild-type (WT) mice. NS101 normalized the spine elimination rate in P301S mice, restoring the net spine count to levels comparable to WT mice. NS101 treatment enhanced the frequency of mEPSCs and fEPSPs in the hippocampal synapses of APP/PS1 mice, leading to improved cognitive function. The increases in plasma FAM19A5 levels upon systemic NS101 administration suggest that the antibody effectively engages its target and facilitates the transport of FAM19A5 from the brain.

**Conclusions:**

This study demonstrated that inhibiting FAM19A5 function with an anti-FAM19A5 antibody restores synaptic integrity and enhances cognitive function in AD, suggesting a novel therapeutic strategy for AD.

**Trial registration:**

https://clinicaltrials.gov/study/NCT05143463, Identifier: NCT05143463, Release date: 3 December 2021.

**Supplementary Information:**

The online version contains supplementary material available at 10.1186/s13195-025-01813-8.

## Introduction

FAM19A5 (family with sequence similarity 19 member A5, also known as TAFA5) is a secretory protein primarily detected in neurons of the central and peripheral nervous systems [1]. Studies utilizing *FAM19A5* promoter-driven LacZ expression in conjugation with qRT‒PCR analysis have demonstrated that *FAM19A5* is highly expressed in the central nervous system, with significantly lower expression in peripheral tissues such as adipose tissues [2, 3]. These findings are corroborated by single-cell/nuclear RNA-seq data from humans and mice, revealing predominant expression of *FAM19A5* in neurons, moderate expression in astrocytes and oligodendrocyte precursor cells, and minimal expression in oligodendrocytes and microglia [4, 5]. The expression of *FAM19A5* in neural precursor cells during early embryonic development suggests its crucial role in neural growth and circuit establishment [6]. Recent investigations employing *FAM19A5* knockout and knock in mice have consistently shown that FAM19A5 deficiency from birth results in hyperactivity, likely due to aberrant spinogenesis and synapse formation during brain development [7, 8].

The involvement of FAM19A5 in synaptic function is supported by the finding that FAM19A1-4, paralogous proteins of FAM19A5, interact with neurexins, presynaptic adhesion molecules that mediate physical contact between pre-and postsynapses [9]. However, FAM19A5 does not bind to neurexin. The amino acid sequences of the FAM19A family are highly conserved across vertebrates, including humans, but FAM19A5 diverges significantly from the FAM19A1-4 family [10, 11], indicating that FAM19A5 has a distinct, yet synapse-related function in the brain. Therefore, it is proposed that FAM19A5 binds to different synaptic receptors, other than neurexins, and is involved in synapse formation and/or elimination.

Alzheimer’s disease (AD) is a neurodegenerative disorder characterized by the progressive loss of synapses, manifested as an imbalance between synapse formation and elimination, leading to cognitive decline. While there are several therapeutic agents approved for treating AD, they offer only limited symptomatic relief and do not slow the progression of the disease [12]. Recently, antibody-based immunotherapy against amyloid beta (Aβ) has attracted significant attention following its approval by the US FDA [13, 14, 15]. However, the limited efficacy in improving cognitive function is likely due to continued synapse loss even after Aβ clearance [15, 16, 17, 18]. Therefore, a novel approach for restoring lost synapses could be a breakthrough, as synapse loss, a fundamental hallmark of AD, contributes to cognitive deficits [19]. Understanding the mechanisms underlying synapse loss in AD may facilitate the development of more potent treatments [20, 21, 22, 23].

We previously demonstrated that partial depletion of FAM19A5 in amyloidopathy APP/PS1 mice increased lifespan and that intravenous (IV) administration of an anti-FAM19A5 antibody improved cognition in APP/PS1 and 5XFAD mice [24]. These findings suggest that partially depleting or inhibiting FAM19A5 in the AD brain may be beneficial. However, the mechanisms by which FAM19A5 influences synapse regulation remain elusive.

Here, we found that FAM19A5 binds to leucine-rich repeat-containing protein 4B (LRRC4B), a postsynaptic adhesion molecule, contributing to a reduction in synaptic contacts in neurons. Conversely, inhibiting FAM19A5 with the anti-FAM19A5 antibody NS101 increased the synapse number. IV administered NS101 was successfully delivered to the brain, facilitating the dose-dependent transport of brain-derived FAM19A5 to the bloodstream in both rodents and humans. IV administration of NS101 to tauopathy P301S mice restored spine density in the cortex through decreasing the spine elimination rate. NS101 also restored synaptic functionality in the hippocampus of APP/PS1 mice. These structural and functional restorations of synapses likely contributed to enhanced cognitive function in both APP/PS1 and P301S mice. These findings suggest that FAM19A5 acts as a physiological modulator of synaptic balance and highlight the clinical potential of anti-FAM19A5 antibodies as a treatment for AD.

## Methods

### Study approval

The mice were maintained in accordance with institutional policies, and all studies were performed with the approval of the Institutional Animal Care and Use Committee of the Korea University (IACUC, KOREA-2020-0031, KOREA-2020-0037, KOREA-2023-0050 and KOREA-2023-0044). Ethical regulations were followed throughout. Two-photon imaging of the mice was performed in accordance with Finnish and EU animal care regulations. Local authorities (Project Authorization Board, Regional State Administrative Agency for Southern Finland) approved the animal license (ESAVI/31282/2019 and ESAVI/31036/2022) to conduct the procedures described in the study. Mouse behavioral tests were conducted in accordance with the Animal Protection Act (Law No. 4372, partially revised Act No. 13023) and approved by the Animal Experimentation Ethics Committee of KPC Co., Ltd. (Approval No. P191005). The study protocol, involving a single-dose administration to healthy volunteers, was reviewed and approved by the Institutional Review Board (IRB) at Advarra (Pro00057986). All participants provided written informed consent in accordance with the Declaration of Helsinki prior to enrolment in the study. The study adhered to Good Clinical Practice (GCP) guidelines and followed all applicable regulatory requirements for clinical research.

### Vertebrate animals

C57BL/6J mice (strain No. 000664), APP/PS1 [B6. Cg-Tg (APPswe, PSEN1dE9) 85Dbo/J] mice (Strain No. 034832-JAX), and P301S [B6. C3-Tg (Prnp-MAPT*P301S) PS19Vle/J] mice (strain No. 008169) were purchased from The Jackson Laboratory. Sprague‒Dawley rats were obtained from Orient Bio. The mice were housed under temperature-controlled conditions (22–23 °C) with a 12-h light/12-h dark-light cycle (lights on at 8:00 am) and had ad libitum access to standard chow and water. The sample size for this study was designed to meet the minimum requirements according to IACUC guidelines and was determined based on the effect size and sample size reported in previous studies [2, 7, 24]. APP-PS1, P301S mice and their wild-type littermates were randomly assigned to treatment groups by alternating the assignment of their cages to groups.

To minimize confounding effects from hormonal fluctuations, particularly the estrous cycle in females, only male mice were used in the behavioral studies. In order to eliminate the confounding variables associated with hormonal fluctuations and pregnancy, the clinical trial was restricted to male participants. Biological samples from both sexes were used in other experiments and yielded similar results.

### Human samples

Pooled normal human CSF was obtained from Innovative Research, Inc. (IPLA-CSFP) and used for Co-IP experiments to determine the association between FAM19A5 and LRRC4B. Individual human CSF and plasma samples were obtained from BIOIVT & ELEVATING SCIENCE and were used to measure age-dependent FAM19A5 levels in CSF and the basal level of FAM19A5 in plasma, respectively. Additionally, individual human CSF samples were also used to determine total Tau and phospho-Tau (pTau) levels.

### Co-immunoprecipitation

HEK293 cells were transfected with reagents from the Neon™ Transfection Kit (Invitrogen). The primer sets used to generate the constructs for Co-IP are listed in Table S1. For the general Co-IP experiments, the cells were treated with 1 µM FAM19A5 for 30 min. The cells were washed twice with cold phosphate-buffered saline (PBS) and resuspended in lysis buffer containing 20 mM Tris-HCl (pH 7.4), 150 mM NaCl, 0.5% NP40, and a protease and phosphatase inhibitor cocktail (Thermo Scientific). The supernatants were isolated by centrifugation at 12,000 × g at 4 °C for 30 min and then mixed with 30 µL of Protein G Dynabeads (Invitrogen) that had been preincubated with antibodies used for IP. The mixtures were incubated overnight at 4 °C on a rotating mixer. The beads were spun down and washed three times with washing buffer containing 20 mM Tris-HCl (pH 7.4), 500 mM NaCl, and 0.5% NP40. After the addition of sample buffer containing a reducing agent to the beads and boiling at 100 °C for 10 min, the proteins were separated via SDS‒PAGE. Detailed Co-IP protocols for human CSF and mouse brain samples are provided in the supplementary material.

### ELISA

To evaluate both total and phosphorylated tau levels, ELISA kits, such as hTAU Ag (Fujirebio) and PHOSPHO-TAU (Fujirebio), were used according to the manufacturer’s instructions. Detailed ELISA protocols for the measurement of FAM19A5 in biofluids and tissues, the measurement of CSF NS101, the assessment of the binding affinity between FAM19A5 and LRRC4 family proteins, and the examination of the inhibition of FAM19A5-LRRC4B binding are provided in the supplementary material.

### Two-photon live spine imaging

Neurotar, a contract research organization, performed the two-photon imaging experiments and data analysis. Thirty-two female P301S mice (B6;C3-Tg (Prnp-MAPT*P301S) PS19Vle/J; JAX Strain #008169), as well as sixteen female B6C3F1/J (JAX Strain #100010) mice, were anesthetized with ketamine/xylazine and subjected to cranial window implantation. Before surgery, the mice received carprofen (subcutaneous, 5 mg/kg) to relieve pain during and after surgery and dexamethasone (subcutaneous, 2 µg/g) to reduce inflammation. The cranial window was inserted over the somatosensory cortex at the following coordinates: AP − 1.8 and ML − 2.0 from the bregma. A dental drill (HP4–917, Foredom) was used to remove a round (d = 4 mm) piece of skull, and the hole in the bone was covered with a round cover glass (d = 5 mm; Electron Microscopy Sciences, 72296–05). A helicopter-shaped head plate (model 1; Neurotar) was placed over the cover glass and fixed to the skull surface with dental cement (Rapid Repair; Dentsply) mixed with cyanoacrylate glue (Loctite 401; Henkel). An AAV viral vector encoding GFP under the control of the synapsin promoter (AAV9-synapsin-GFP, Signagen, SL100845-Std) was injected intracortically during surgery (300 nanoliters, titer 1:10, depth of injection 800–900 micrometers from the cortical surface) at the following coordinates: AP − 1.8 and ML − 2.0 from the bregma to fluorescently labeled neurons. Of the 48 mice that underwent cranial window implantation, we selected 30 with the clearest windows for imaging, starting three weeks post-surgery. In the fourth week, these selected mice began a four-day habituation protocol to acclimate them to head fixation. Imaging was initiated at the end of this habituation period, corresponding to four weeks post-surgery. We then longitudinally imaged the same brain region of interest (ROI) over 66 days at seven predetermined time points: days 1, 5, 9, 13, 17, 38, and 66.

Seven-month-old P301S mice were imaged with an FV1200MPE two-photon microscope (Olympus, Japan) with a 25X water immersion 1.05 NA objective specially designed for in vivo two-photon imaging. A MaiTai Broad Band DeepSee laser tuned to 900 nm was used for excitation. Emission light was collected using a bandpass filter (515–560 nm). For in vivo imaging sessions, awake animals were head-fixed under a two-photon microscope using the Mobile Home Cage device (Neurotar, Finland). One week prior to the start of imaging, the animals were habituated to the head fixation conditions for four days.

In vivo two-photon imaging for analyzing dendritic spine dynamics was performed under conditions consistent with those described in a previously established protocol [25]. A single cranial window per mouse, and a single field of view was imaged 7 times per time point. The images were acquired in z-stack mode with a z-interval of 1 μm, yielding a total of 71 optical sections. The original lateral resolution was 0.313-µm-per-pixel x-y resolution. For analysis purposes, the images were upscaled post-acquisition to 0.104-µm-per-pixel to facilitate accurate spine quantification. The number of dendrites analyzed per field ranged from 16 to 36 branches (mean ± SD: 25.2 ± 5.5 per field).

After image acquisition, the images were processed and analyzed using ImageJ (NIH). First, shifts between time points in the x, y, and z directions were compensated using Neurotar’s scripts in ImageJ. Spine turnover analysis was performed by a scientist completely blinded to the identity of the treatment groups. Individual spines were tracked manually by visual identification in 3D stacks of images collected at all time points. Protrusions of at least 5 pixels from the dendritic shaft were considered candidate spines. After identification of a candidate spine, the data analyst examined 10 adjacent slices in both directions to determine other explanations for the protrusion. If no other explanation was found, the spine was counted in the analysis. At every time point, each spine was assigned a specific code, either 1 (present) or 0 (absent). If, in some instances, the image quality was not sufficiently sharp for spine identification at a specific time point, the spine was assigned a code − 1 and excluded from further analysis. Dendritic spine dynamics were analyzed by categorizing spines into three groups according to established definitions [25, 26]. Data were pooled for each treatment group, and statistical analysis was performed on the following parameters: Formation: the fraction of spines that were newly formed (“gain”). Elimination: the fraction of spines that disappeared (“loss”). Stable: the fraction of spines that did not change (“stable”). The fraction of gained and lost spines was calculated by normalizing the number of gained, lost or stable dendritic spines to the enumerated total dendritic spine numbers. The spine formation and elimination were then normalized to the average of corresponding values calculated from the images acquired before drug administration.

### Counting of dendritic spines

Dendritic spines were imaged from layers II/III of the prefrontal cortex and the CA1 of the hippocampus using a 63x oil-immersion lens with 3x digital zoom. Segments of 30 μm long basal dendrites were sampled. A total of 160 images were obtained from the brain Sect. (80 μm thick at 0.5 μm intervals). Maximum intensity projection (MIP) images were obtained from a total of 160 images and processed with ImageJ software (NIH). Five MIP images per subject were selected for quantification. The dendritic spine counter in ImageJ was used for dendritic spine counting and classification. At least 15 dendrites per subject were measured, and spines were classified as mushroom, stubby, or thin using the criteria [27, 28].

### Quantification and statistical analysis

All the statistical analyses were performed using GraphPad Prism 8 with 95% confidence. All the data are presented as the mean ± SEM. All the details of the statistical tests and results are reported in the figure legends.

## Results

### FAM19A5 interacts with LRRC4B

**Fig. 1:**
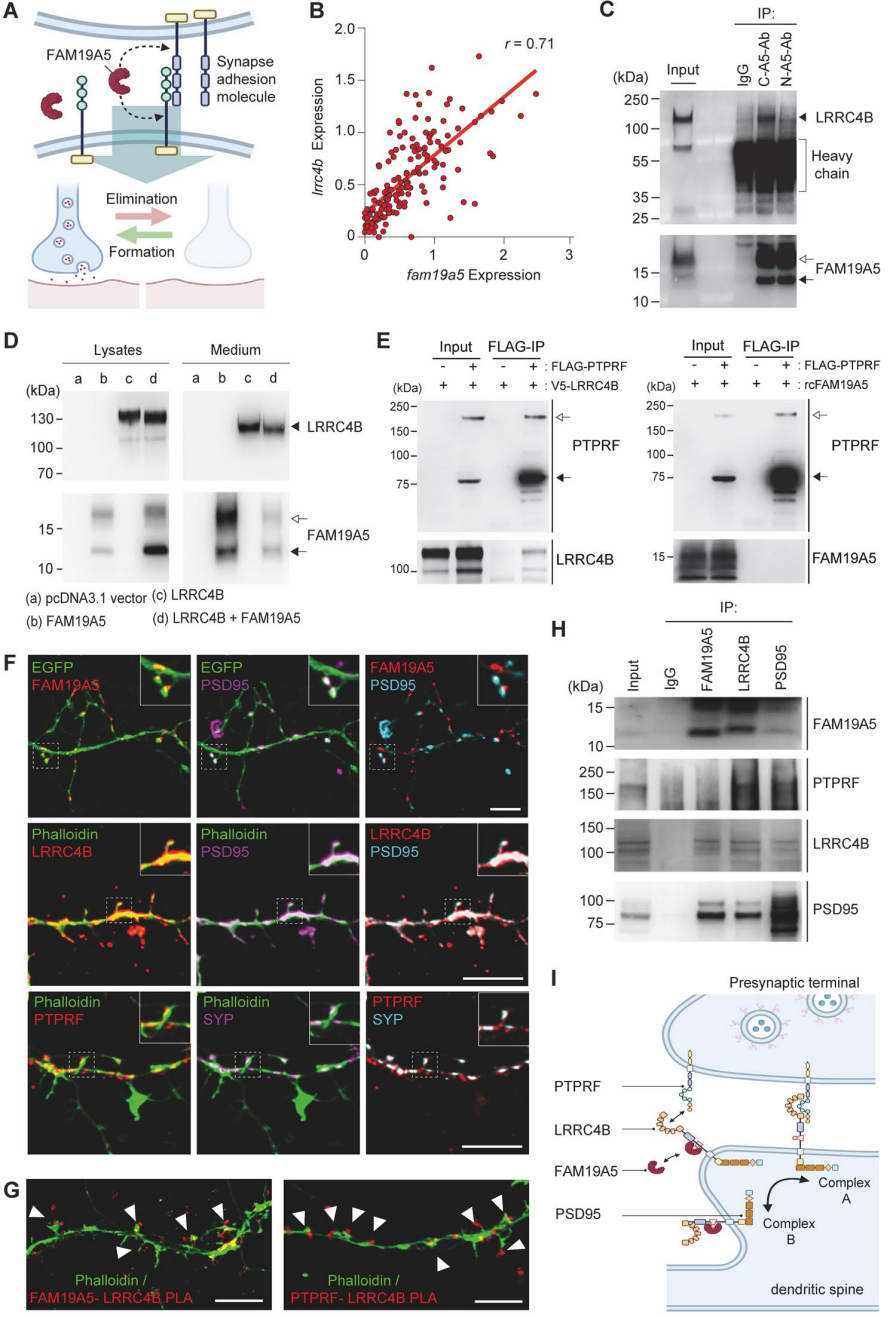
FAM19A5 binds to LRRC4B. (**A**) Illustration of the potential mechanisms of synapse regulation. FAM19A5 binding to synapse adhesion molecules can induce either synapse elimination or formation. (**B**) Correlations between the RNA transcript levels of *lrrc4b* and *fam19a5* in 168 neuron types. Correlation coefficient; *r* = 0.71, *P* < 0.0001. (**C**) Co-IP of human CSF using anti-FAM19A5 antibodies (C-A5-Ab or N-A5-Ab). Solid arrowhead: LRRC4B, open arrow: glycosylated FAM19A5, solid arrow: nonglycosylated FAM19A5. The band for the antibody’s heavy chain was marked with a single parenthesis. (**D**) Immunoblots of LRRC4B and FAM19A5 in culture lysate and medium from HEK293 cells coexpressing LRRC4B and FAM19A5. Solid arrowhead: LRRC4B, open arrow: glycosylated FAM19A5, solid arrow: nonglycosylated FAM19A5. (**E**) Co-IP was performed using HEK293 cells coexpressing LRRC4B-V5 and PTPRF-Flag (left). Recombinant FAM19A5 was added to investigate the interaction with PTPRF (right). Open arrow: full-length of PTPRF, solid arrow: p subunit of PTPRF. (**F**) Representative image of a dendritic spine in green from a cultured hippocampal neuron at 13 DIV showing the localization of the synaptic proteins FAM19A5 (red, overexpressed), PTPRF (red), SYP (magenta/cyan), LRRC4B (red), and PSD95 (magenta/cyan). The dotted square area in each image is magnified in the upper right corner. Scale bar, 1 μm. (**G**) Representative image of a FAM19A5-LRRC4B and PTPRF-LRRC4B interaction on the dendritic spines of cultured hippocampal neurons, as demonstrated by the PLA. Scale bar, 5 μm. (**H**) Co-IP using lysates prepared from the mouse cerebral cortex to investigate the interaction between FAM19A5, PTPRF, LRRC4B and PSD95. (**I**) Illustration of the potential regulation of synaptic adhesion molecules by FAM19A5. The formation of complexes A and B, consisting of PSD95-LRRC4B-PTPRF and PSD95-LRRC4B-FAM19A5, respectively, is regulated by FAM19A5

We hypothesized that FAM19A5, as a synapse-regulating factor, may interact with synaptic adhesion molecules other than neurexins [9] to fine-tune the synaptic balance (Fig. 1A). To explore potential binding molecules for FAM19A5, we investigated RNA expression patterns from 168 types of neurons in the mouse brain single-cell RNA-seq database [4], comparing their correlation with *fam19a5* to narrow down candidates. We found that the expression of *lrrc4b*, a postsynaptic adhesion molecule, was highly correlated with that of *fam19a5* (Fig. 1B and Supplementary Fig. 1). Given their similar expression patterns and their secretion into the cerebrospinal fluid (CSF) [29, 30, 31], it is plausible that FAM19A5 and LRRC4B may exist in a complex form in the CSF. Co-immunoprecipitation (Co-IP) with human CSF using monoclonal antibodies against FAM19A5 revealed that LRRC4B was co-immunoprecipitated with an antibody that binds to the C-terminal region of FAM19A5, suggesting that FAM19A5 and LRRC4B may form a complex in the brain (Fig. 1C). Western blot revealed two distinct forms of FAM19A5, attributed to posttranslational modifications such as glycosylation [2]. The FAM19A5-LRRC4B interaction was further confirmed by Co-IP assays using HEK293 cells expressing LRRC4B after treatment with recombinant FAM19A5 (Supplementary Fig. 2).

Additional Co-IP experiments using HEK293 cells expressing FAM19A5 and LRRC4B revealed a lower molecular weight form of LRRC4B in the culture media compared to LRRC4B present in the cell lysate. LRRC4B detected in the medium is likely a secreted form, cleaved at the juxta-membrane region of full-length LRRC4B [32]. Upon coexpressing LRRC4B, the concentration of secreted FAM19A5 in the medium decreased, while its concentration in the lysates markedly increased, further supporting the formation of a FAM19A5-LRRC4B complex (Fig. 1D). LRRC4B is known to interact with protein tyrosine phosphatase receptor type F (PTPRF), a presynaptic cell adhesion protein, facilitating the bridging of pre- and post-synapses [33, 34]. Co-IP experiments using HEK293 cells expressing PTPRF and LRRC4B confirmed the interaction between these two molecules. However, no interaction was observed between PTPRF and FAM19A5 (Fig. 1E).

To further investigate potential interactions between these molecules under physiological conditions, immunocytochemistry was performed on cultured mouse hippocampal neurons. FAM19A5 immuno-positive signals were detected in dendritic spines where postsynaptic density protein 95 (PSD95) is expressed, specifically in the spine neck. LRRC4B was largely colocalized with PSD95 in postsynaptic spines along dendrites (Fig. 1F). Both the FAM19A5 and LRRC4B signals were scarcely detected in synaptophysin (SYP)-positive presynaptic structures (Supplementary Fig. 3). In contrast, PTPRF-immunopositive signals were observed in SYP-positive areas but not in PSD95-positive dendritic spines (Fig. 1F and Supplementary Fig. 3). A proximity ligation assay confirmed a direct interaction between FAM19A5 and LRRC4B near dendritic spines (Fig. 1G and Supplementary Fig. 4).

Additionally, Co-IP using lysates prepared from the mouse cerebral cortex revealed that FAM19A5 forms a complex with LRRC4B and PSD95 but not with PTPRF. Interestingly, LRRC4B also forms a complex with PTPRF, independent of the FAM19A5-LRRC4B-PSD95 complex. PSD95 forms complexes with both LRRC4B and PTPRF, with FAM19A5 weakly bound to this complex (Fig. 1H). These findings suggest the formation of two distinct complexes at the synapse: one consisting of FAM19A5-LRRC4B-PSD95 and another without FAM19A5, consisting of PTPRF-LRRC4B-PSD95. These results suggest that FAM19A5 may disrupt the PTPRF-LRRC4B complex by binding to LRRC4B (Fig. 1I).

### FAM19A5 binds to the FB domain of LRRC4B

**Fig. 2:**
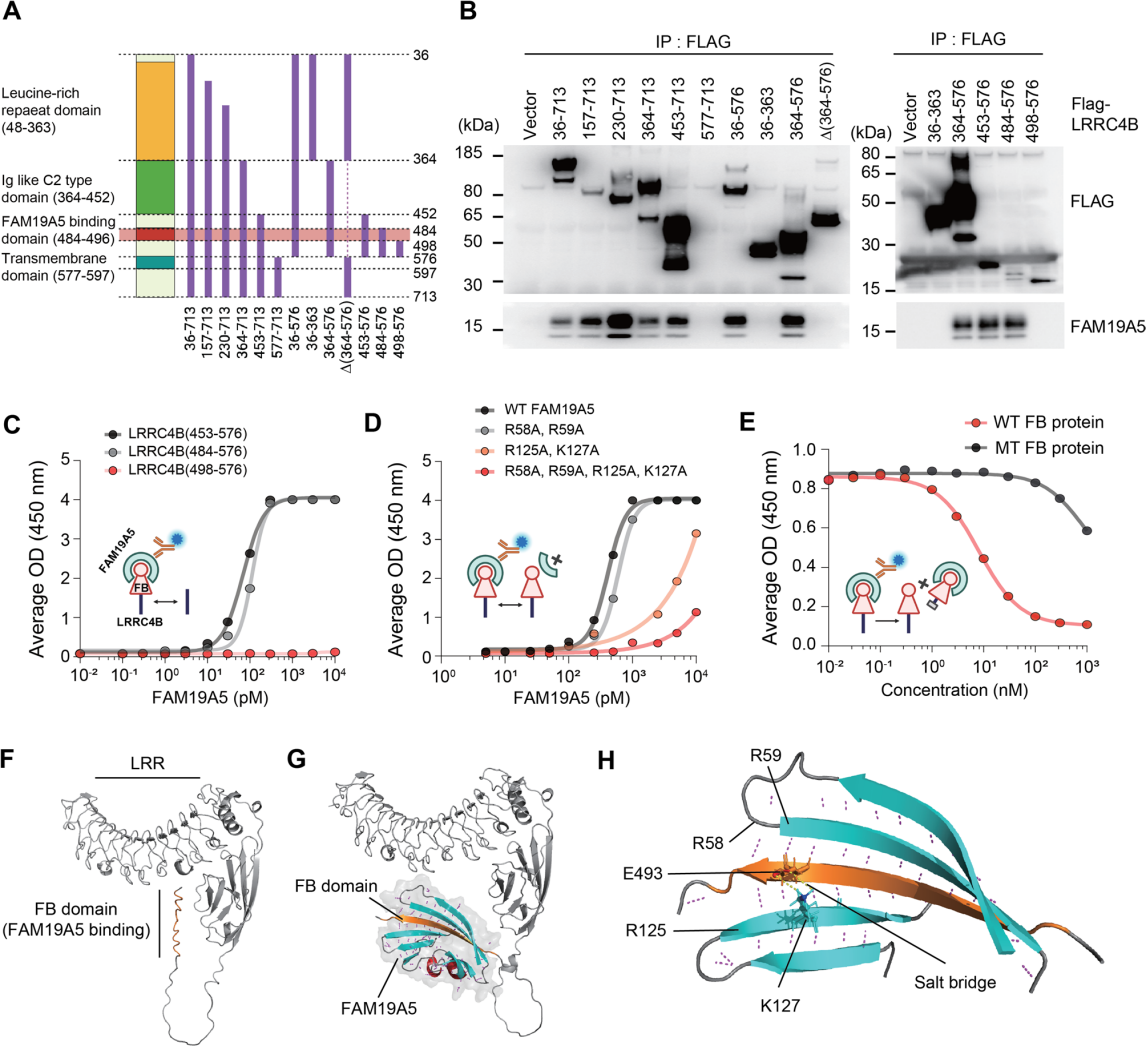
Characterization of FAM19A5-LRRC4B binding. (**A**) Domain architecture of the LRRC4B deletion constructs. (**B**) Co-IP of interactions between FAM19A5 and LRRC4B deletion constructs. (**C**) The binding affinity of LRRC4B for FAM19A5 was determined via ELISA (inserted, captured by LRRC4B-hFc, detected by HRP-conjugated C-A5-Ab). The EC_50_ of LRRC4B(453–576)-hFc = 68.5 pM, and the EC_50_ of LRRC4B(484–576)-hFc = 112.1 pM. (**D**) Binding affinity of wild-type (WT) or mutant (MT) FAM19A5 proteins (R58A, R59A; R125A, K127A) to LRRC4B measured via ELISA (inserted, captured by His-TEV LRRC4B, detected by HRP-conjugated C-A5-Ab). The EC_50_ of WT FAM19A5 = 409.5 pM, and the EC_50_ of MT FAM19A5 (R58A, R59A) = 594.8 pM. (**E**) Inhibition of FAM19A5-LRRC4B binding by WT LRRC4B(453–576)-hFc or MT LRRC4B(453–576, T488A, T489A)-hFc FB-containing proteins determined via ELISA (inserted, captured by LRRC4B(36–576)-hFc, detected by HRP-conjugated C-A5-Ab). The IC_50_ of the WT FB-containing protein was 8.1 nM. (**F**) In silico model of LRRC4B, including the leucine-rich repeat (LRR), immunoglobulin C (IgC), and FB domains (orange). (**G**) In silico model of the LRRC4B-FAM19A5 complex. The FAM19A5 structures (gray surface) are depicted as α-helices (red) and β-strands (cyan). (**H**) Model showing the salt bridge between the side chains of E493 (orange) and K127 (cyan). Hydrogen bonds are represented by dashed lines. The predicted key residues of FAM19A5 involved in binding to the FB domain (484–498, orange) are R58, R59, R125, and K127

To determine which domain of LRRC4B interacts with FAM19A5, we generated a series of LRRC4B fragments (Fig. 2A) and performed Co-IP assays. All constructs containing the 484–498 amino acid (aa) sequence of LRRC4B bound to FAM19A5, while deletion of the 484–498 aa sequence abolished FAM19A5 binding (Fig. 2B and Supplementary Fig. 5A). ELISA further confirmed this interaction, showing high affinity for LRRC4B fragments containing the amino acid range of 484–496 (EC_50_ values were 68.5 pM for the sequence 453–576 and 112.1 pM for 484–576). No binding was evident without the sequence (Fig. 2C). These results demonstrate that the LRRC4B sequence in the range of residues 484–498 acts as a FAM19A5 binding domain, termed the FB domain. Surface plasmon resonance (SPR) measurements revealed a pronounced binding affinity between FAM19A5 and LRRC4B(453–576), with an equilibrium dissociation constant (K_D_) of 32 pM (Supplementary Fig. 5B). This affinity notably stands out from that reported for other synaptic adhesion molecules [35].

To elucidate essential residues within the FB domain, we investigated the binding free energy and compared its changes after the substitution of each residue with Ala. All the residues, except Thr494 and Leu495, played vital roles in binding (Supplementary Fig. 5C), further supported by the ELISA results (Supplementary Fig. 5D). Additionally, residues Arg58, Arg59, Arg125, and Lys127 of FAM19A5 are predicted to be the key residues involved in binding to LRRC4B (Supplementary Fig. 5E). Subsequent ELISAs demonstrated that Arg125 and Lys127 were significant for LRRC4B binding, while Arg58 and Arg59 contributed moderately to the interaction (Fig. 2D). The amino acid sequence of the FB domain is highly conserved across vertebrate species, including humans (Supplementary Fig. 5F), implying a crucial function of the FAM19A5-LRRC4B complex in the brain. To further validate the FAM19A5-FB interaction, we measured FAM19A5-LRRC4B binding in the presence of wild-type (WT) or mutant (MT) FB-containing protein (LRRC4B[453–576, T488A, T489A]-hFc), which does not bind to FAM19A5. The WT FB-containing protein effectively inhibited the binding of FAM19A5 to LRRC4B in a dose-dependent manner, whereas the MT FB-containing protein had minimal impact (Fig. 2E). These competition experiments further support the specific interaction between the FB domain of LRRC4B and FAM19A5.

Using AlphaFold2 and RoseTTAFold [36, 37, 38], we performed 3D modelling of the FAM19A5-LRRC4B complex to delve into their structural and molecular interactions. Complex modelling showed that these proteins interact through the key residues of FAM19A5 and the FB domain of LRRC4B, supporting the results of the biochemical analyses shown in Fig. 2A, B. The sequence of FB, exhibiting a disordered structure prior to binding with FAM19A5, was transformed into a β-strand. The β-strand then interacted with the β-strands of FAM19A5, primarily via hydrogen bonds (Fig. 2F and G). Furthermore, predictions indicated the formation of a salt bridge between LRRC4B Glu493 and FAM19A5 Lys127, likely further stabilizing the complex structure (Fig. 2H).

### FAM19A5 binding to LRRC4B leads to the dissociation of synaptic contacts

**Fig. 3:**
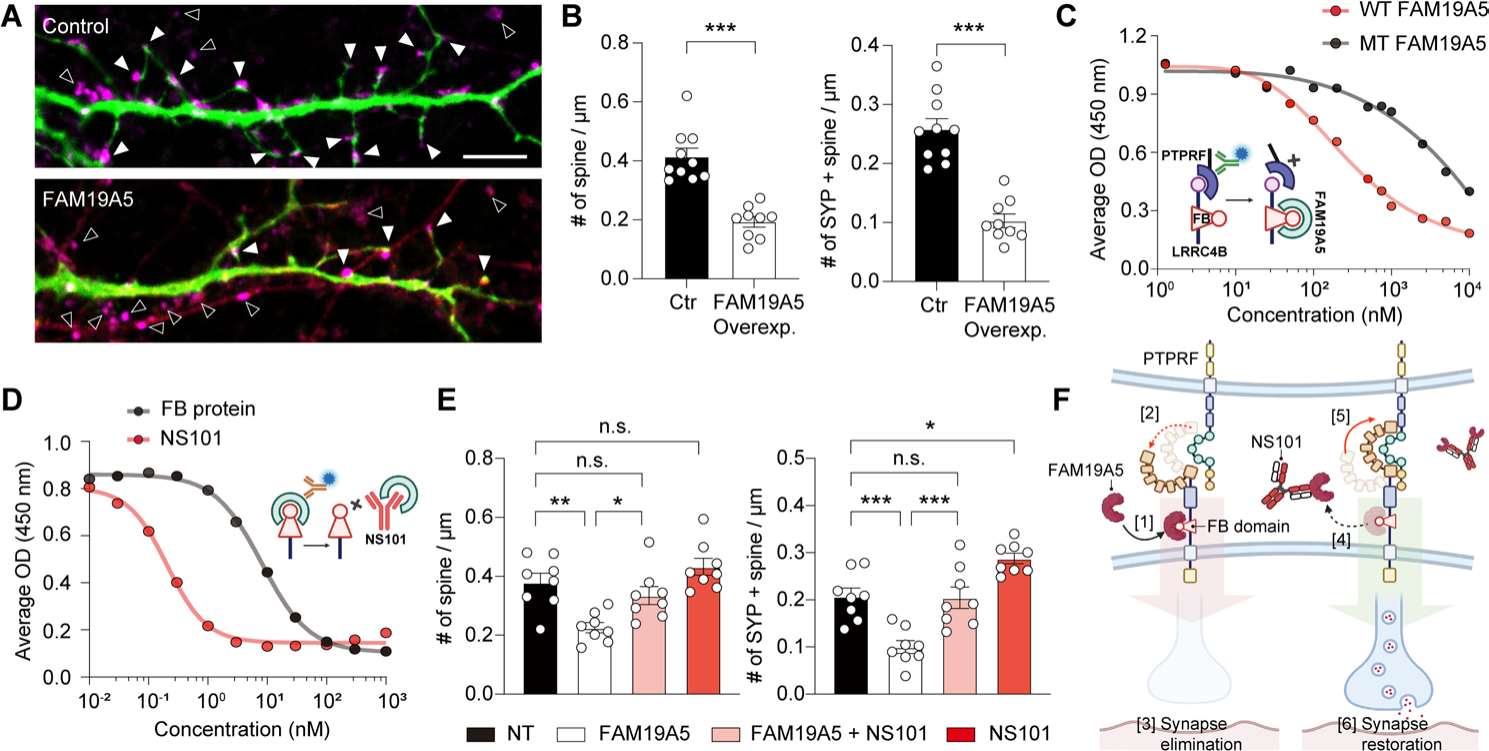
FAM19A5 binding to LRRC4B leads to the dissociation of synaptic contacts. (**A**) Representative image of dendritic spines on cultured hippocampal neurons transfected with either the EGFP alone (control, green) or EGFP and FAM19A5 (red). SYP signals (magenta) contacting the spine are marked with solid arrowheads, while those without spine contact are marked with open arrowheads. (**B**) Quantification of the number of spines and spines in contact with SYP (*n* = 7 per group). Data are presented as the mean ± SEM. Unpaired ttest was used to calculate P values (number of spines: t_17_ = 6.415, *P* < 0.0001; number of spines in contact with SYP: t_17_ = 7.065, *P* < 0.0001). **P < 0.01 (*P* = 0.0051), ***P < 0.001. (**C**) Dissociation of the LRRC4B-PTPRF complex in the presence of WT FAM19A5 or MT FAM19A5 [R58A, R59A, R125A, K127A] determined via ELISA (inserted, captured by His-TEV LRRC4B, detected by HRP-conjugated anti-human IgG Fc). The IC_50_ of WT FAM19A5 = 281 nM. (**D**) Inhibition of FAM19A5-LRRC4B binding by NS101 or FB-containing proteins determined via ELISA (inserted, captured by LRRC4B(36–576)-hFc, detected by HRP-conjugated C-A5-Ab). The IC_50_ of NS101 was 205.0 pM, and the IC_50_ of the FB protein was 8.1 nM. (**E**) Quantification of the number of spines, and spines in contact with SYP (*n* = 7 per group) in hippocampal neurons treated with NT, FAM19A5 (50 nM), or NS101 (50 nM) or cotreated with FAM19A5 and NS101 (50 nM). Data are presented as the mean ± SEM. One-way ANOVA followed by Tukey’s multiple comparison test was used to calculate P values (number of spines: F_3, 28_ = 0.4023, *P* = 0.7524; number of spines in contact with SYP: F_3, 28_ = 0.9126, *P* = 0.4475). ***P < 0.001. (**F**) Schematic illustration depicting the mechanism of FAM19A5-mediated synapse destabilization and NS101-induced restoration. FAM19A5 binding to the FB domain of LRRC4B disrupts the PTPRF-LRRC4B complex, leading to synaptic structural instability [1→2], and eventual synapse elimination [3]. Conversely, NS101 inhibits FAM19A5 binding to LRRC4B [4], allowing the formation of the PTPRF-LRRC4B complex [5]. This restabilizes the synaptic structure, resulting in synapse restoration [6]

Mouse primary hippocampal neurons endogenously expressed high levels of FAM19A5, LRRC4B, and PTPRF transcripts since their early stage of development (Supplementary Fig. 6). To investigate the role of FAM19A5 in synapse formation or elimination, we overexpressed FAM19A5 in hippocampal neurons. Neurons were coexpressed with EGFP to visualize dendritic spines, and synaptic contacts were quantified by counting those colocalized with the presynaptic marker SYP. Overexpression of FAM19A5 significantly reduced the density of spines, particularly those in contact with SYP, compared to neurons transfected with EGFP alone (Fig. 3A and B).

Given that LRRC4B forms a transsynaptic connection with PTPRF [34], we postulated that FAM19A5 binding to LRRC4B may induce a conformational change in the LRRC4B-PTPRF complex, resulting in synapse reduction. To test this hypothesis, we performed ELISAs to quantitatively evaluate LRRC4B-PTPRF binding. WT FAM19A5 significantly decreased LRRC4B-PTPRF binding in a dose-dependent manner, whereas MT FAM19A5[R58A, R59A, R125A, K127A], lacking binding affinity for the FB domain of LRRC4B, only partially inhibited this binding (Fig. 3C, IC_50_ of WT FAM19A5: 281 nM). Additional Co-IP experiments also confirmed a dose-dependent decrease in LRRC4B-PTPRF binding upon increasing FAM19A5 concentration (Supplementary Fig. 7). These results demonstrated that FAM19A5 can dissociate the LRRC4B-PTPRF complex.

Building upon this discovery, we hypothesized that conversely, detaching FAM19A5 bound to LRRC4B can promote synapse restoration. For specific targeting of FAM19A5 in the brain, we developed a monoclonal antibody against FAM19A5 called NS101. The Fc region of NS101 was further engineered to prevent interaction with Fcγ receptors on immune cells, potentially mitigating the risk of antibody-dependent cellular cytotoxicity and complement-dependent cytotoxicity [39]. Among the FAM19A family members, NS101 exhibited specific binding to FAM19A5 (Supplementary Fig. 8A) with a strong affinity (K_D_ =111 pM, Supplementary Fig. 8B). ELISAs using FAM19A5 fragments demonstrated that the epitopes for NS101 were Arg52, Pro57, Arg58 and Arg59 (Supplementary Fig. 8C). Notably, Arg58 and Arg59 are key residues of FAM19A5 for its binding to LRRC4B, indicating that NS101 can inhibit FAM19A5-LRRC4B complex formation by competitively binding to these key residues. Owing to this high affinity, NS101 dissociated the FAM19A5-LRRC4B complex more effectively than the synthetic FB-containing protein (Fig. 3D, IC_50_ for NS101: 0.2 nM vs. IC_50_ for FB-containing protein: 8 nM).

To examine the effect of NS101 on FAM19A5-mediated synapse elimination, primary hippocampal neurons were treated with NS101. Treatment of neurons with FAM19A5 decreased spine density. However, when NS101 was administered together with FAM19A5, NS101 effectively blocked the spine-reducing effect of FAM19A5. Treatment with NS101 alone increased SYP-positive spine density (Fig. 3E). NS101 likely dissociates FAM19A5 from LRRC4B on neurons, as evidenced by a significant increase in FAM19A5 levels in the culture media following treatment with NS101 (Supplementary Fig. 9). Similarly, treatment of hippocampal neurons with WT FB-containing protein, but not with MT FB-containing protein, significantly increased the intensity and colocalization of SYP and PSD95 compared to those in the control group (Supplementary Fig. 10).

Collectively, these findings suggest that FAM19A5, by specifically binding to LRRC4B, disrupts the LRRC4B-PTPRF complex within synapses, contributing to synapse elimination. However, removing FAM19A5 bound to LRRC4B via NS101 can preserve the complex and trigger the reassociation of LRRC4B-PTPRF, thereby restoring synapses (Fig. 3F).

### NS101 enters the brain and transports brain FAM19A5 into the peripheral circulation

Building upon the established role of FAM19A5 as a physiological synaptolytic factor, we hypothesized that amyloid plaques and tau tangles in AD act as pathological drivers of excessive synapse elimination, tilting the balance toward detrimental synapse loss. Recent RNA sequencing analysis of brain tissue from AD patients has revealed increased levels of *FAM19A5* mRNA [5, 40, 41], suggesting a potential contribution to the excessive synaptic loss. To investigate the potential association of FAM19A5 with AD pathology, we measured the concentrations of FAM19A5 and tau proteins in CSF collected from humans of different ages. FAM19A5 levels in human CSF increased with age (Supplementary Fig. 11A). Furthermore, the levels of CSF tau proteins, including pTau, the primary component of neurofibrillary tangles in AD [42, 43] also increased in parallel with the CSF FAM19A5 levels (Supplementary Fig. 11B). Moreover, FAM19A5 was co-immunoprecipitated with early-stage pTau including S202/T205, T181, and T217 [44, 45], but not with T231 or S396, suggesting a role of FAM19A5 in AD progression (Supplementary Fig. 11C).

**Fig. 4:**
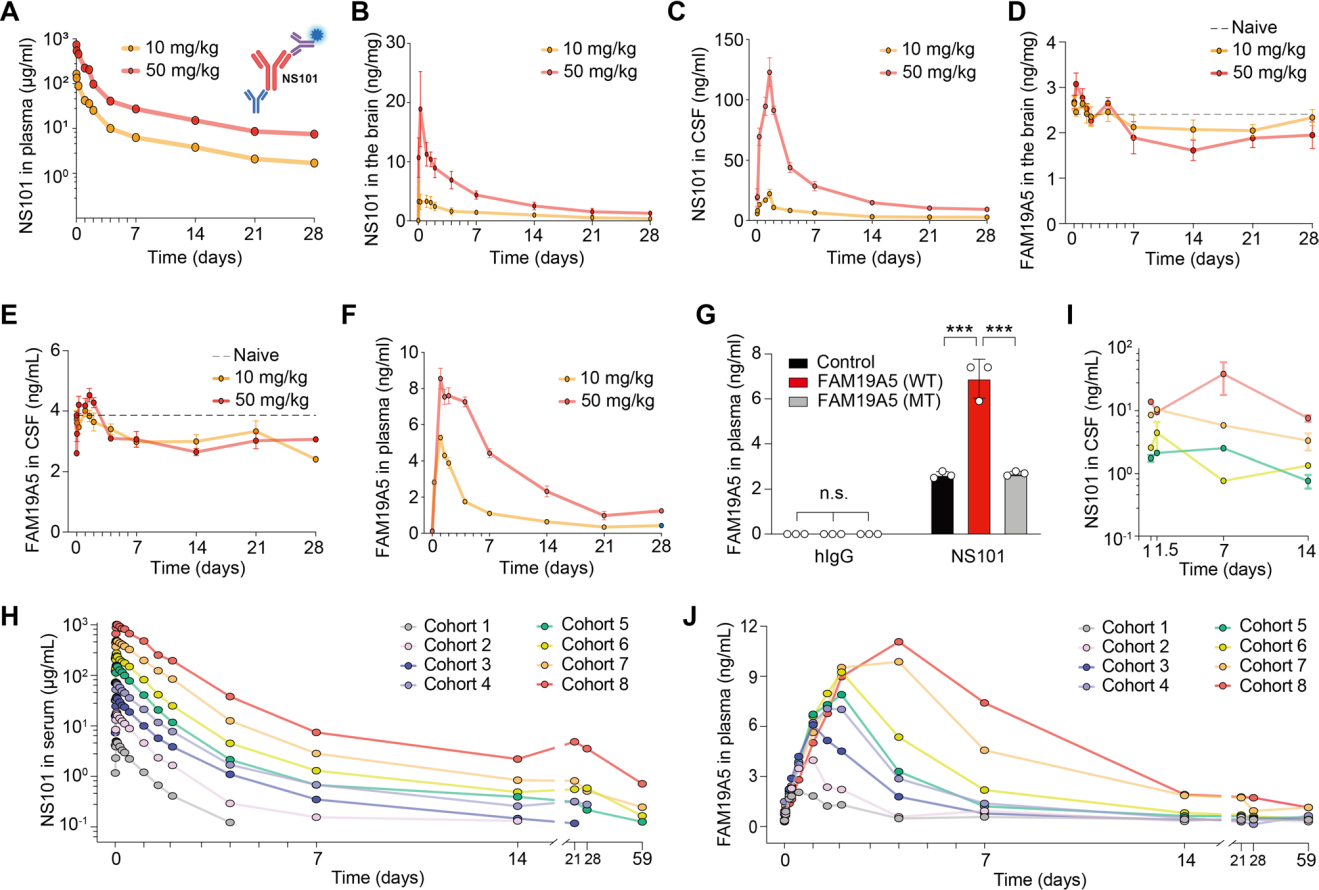
NS101, a potent monoclonal antibody against FAM19A5. (**A**) Plasma NS101 levels at the indicated time points after a single IV administration of 10 mg/kg or 50 mg/kg NS101 determined via ELISA (inserted, captured by rabbit anti-human IgG Fc, detected by HRP-conjugated hIgG kappa light chain antibody). NS101 levels in the (**B**) rat brain lysate and (**C**) rat CSF at the indicated time points after a single IV administration of 10 or 50 mg/kg NS101 determined via ELISA (captured by rabbit anti-human IgG heavy chain, detected by HRP-conjugated hIgG kappa light chain antibody). (**D**) FAM19A5 levels in the rat brain after a single administration of 10 or 50 mg/kg NS101 determined via ELISA (captured by S-A5-Ab, detected by HRP-conjugated SS01). (**E**) Brain-secreted FAM19A5 levels in rat CSF 48 h after a single IV administration of 10 or 50 mg/kg NS101 (captured by S-A5-Ab, detected by HRP-conjugated SS01). (**F**) Brain-secreted FAM19A5 levels in rat blood plasma at the indicated time points after a single IV administration of 10 or 50 mg/kg NS101, as determined via ELISA (inserted, captured by His-TEV hLRRC4B(453–576), detected by HRP-conjugated C-A5-Ab). (**G**) Plasma FAM19A5 levels after brain infusion of WT and MT FAM19A5 (R58A, R59A). One day after the infusion, the mice were IV administered hIgG or 10 mg/kg NS101. ELISA was performed using His-TEV LRRC4B and HRP-conjugated SS01 (*n* = 3 per group). One-way ANOVA followed by Tukey’s multiple comparison test was used to calculate P values (F_5, 12_ = 169.5, *P* < 0.001). n.s., not significant; ***P < 0.001. NS101 levels in (**H**) human serum and (**I**) human CSF at the indicated time points after a single IV administration of 0.25 to 48 mg/kg were determined via ELISA (captured by HIS-TEV FAM19A5 and rabbit anti-NS101, detected by sulfo-tag anti-rabbit antibody). (**J**) Brain-secreted FAM19A5 levels in human blood plasma at the indicated time points after administration determined by ELISA (captured by S-A5-Ab, detected by HRP-conjugated SS01). Data are presented as the mean ± SEM

The potential role of FAM19A5 in AD progression suggests that targeting FAM19A5 with NS101 may restore the synaptic balance by reducing excessive synapse elimination. We examined whether IV administered NS101 can enter the brain and bind to FAM19A5. To evaluate the pharmacokinetics of NS101, we monitored its concentration in blood over time following a single IV administration in rats, which are well suited for longitudinal studies due to their capacity for repeated blood sampling. NS101 was gradually excreted over a span of 28 days, with a half-life of 7.5 days for a dose of 10 mg/kg and 11.7 days for a dose of 50 mg/kg (Fig. 4A). The area under the curve (AUC) revealed a dose-dependent increase in systemic exposure to NS101 (10,336.3 h·µg/mL for 10 mg/kg and 54,415.0 h·µg/mL for 50 mg/kg). Similar to other clinical antibodies [46], prolonged systemic exposure to NS101 facilitated its delivery to the brain across the blood-brain barrier (BBB). The amount of NS101 delivered to the brain was dose- and time-dependent. NS101 peaked in the rat brain 6 h post-injection and declined slowly over 28 days, with a half-life of 11.0 (10 mg/kg) and 8.3 (50 mg/kg) days (Fig. 4B). NS101 in the brain was either excreted directly to the blood vessels or transported via the CSF. NS101 reached peak concentration in the rat CSF at 36 h, exhibiting a dose-dependent increase (Fig. 4C).

To evaluate the target engagement of NS101, we quantified the alterations in FAM19A5 levels in the rat brain. After an initial increase during the 4-day period following a single administration of NS101, the FAM19A5 levels in the brain decreased over 28 days (Fig. 4D). Similarly, after an initial fluctuation, CSF FAM19A5 levels were also decreased (Fig. 4E). Plasma FAM19A5 levels, negligible under normal condition [2], increased sharply after NS101 administration for the first 2 days and then gradually decreased over 28 days in a dose-dependent manner (Fig. 4F). This suggests that FAM19A5 may be released from the brain upon interaction with NS101. We confirmed that the brain is the source of plasma FAM19A5 by infusing the mouse brain with recombinant FAM19A5. Brain infusion did not alter the baseline plasma levels of FAM19A5. However, NS101 treatment significantly elevated plasma FAM19A5 levels in the FAM19A5-infused group compared to controls, while infusion with MT FAM19A5 did not (Fig. 4G). These findings suggest that the binding of FAM19A5 to NS101 facilitates its clearance from the brain, resulting in elevated plasma FAM19A5 levels and a consequent decrease in brain FAM19A5 levels.

We then investigated the safety, pharmacokinetics, and target engagement of NS101 in a phase 1 clinical trial involving 64 healthy subjects. The subjects were divided into 8 groups, with 2 subjects per group receiving a placebo. The antibody dose was set at 0.25-48.0 mg/kg. The concentration of IV infused NS101 in the serum increased rapidly, with a median T_max_ of 0.98 to 1.74 h across the doses. Subsequently, NS101 levels slowly decreased over 60 days (Fig. 4H). Antibodies delivered to the brain across the BBB are secreted into the CSF. NS101 secreted into the CSF was detected in the 6 mg/kg dose group and above, and the amount of NS101 detected tended to increase in proportion to the dose (Fig. 4I). In the placebo group, only approximately 0.3 ng/ml plasma FAM19A5 was detected. In contrast, the peak concentration and time to maximum concentration of plasma FAM19A5 significantly increased in a dose-dependent manner following the administration of NS101, as determined by the median time to maximum effect (median TE_max_ of 11.96 to 95.55 h). At concentrations greater than 6 mg/kg, NS101 did not distinctly increase the peak concentration of FAM19A5 but increased the total amount of plasma FAM19A5 in a dose-dependent manner (the AUC ranged from 707843.52 to 7730521.14 h·pg/mL) (Fig. 4J). No dose-related adverse events, including serious side effects and death, were found in any of the experimental groups. These results demonstrate that NS101 can be delivered to the brain and target FAM19A5 in humans in the same way.

### NS101 prevents synapse loss, increasing the number of functional synapses in mouse models of AD

**Fig. 5:**
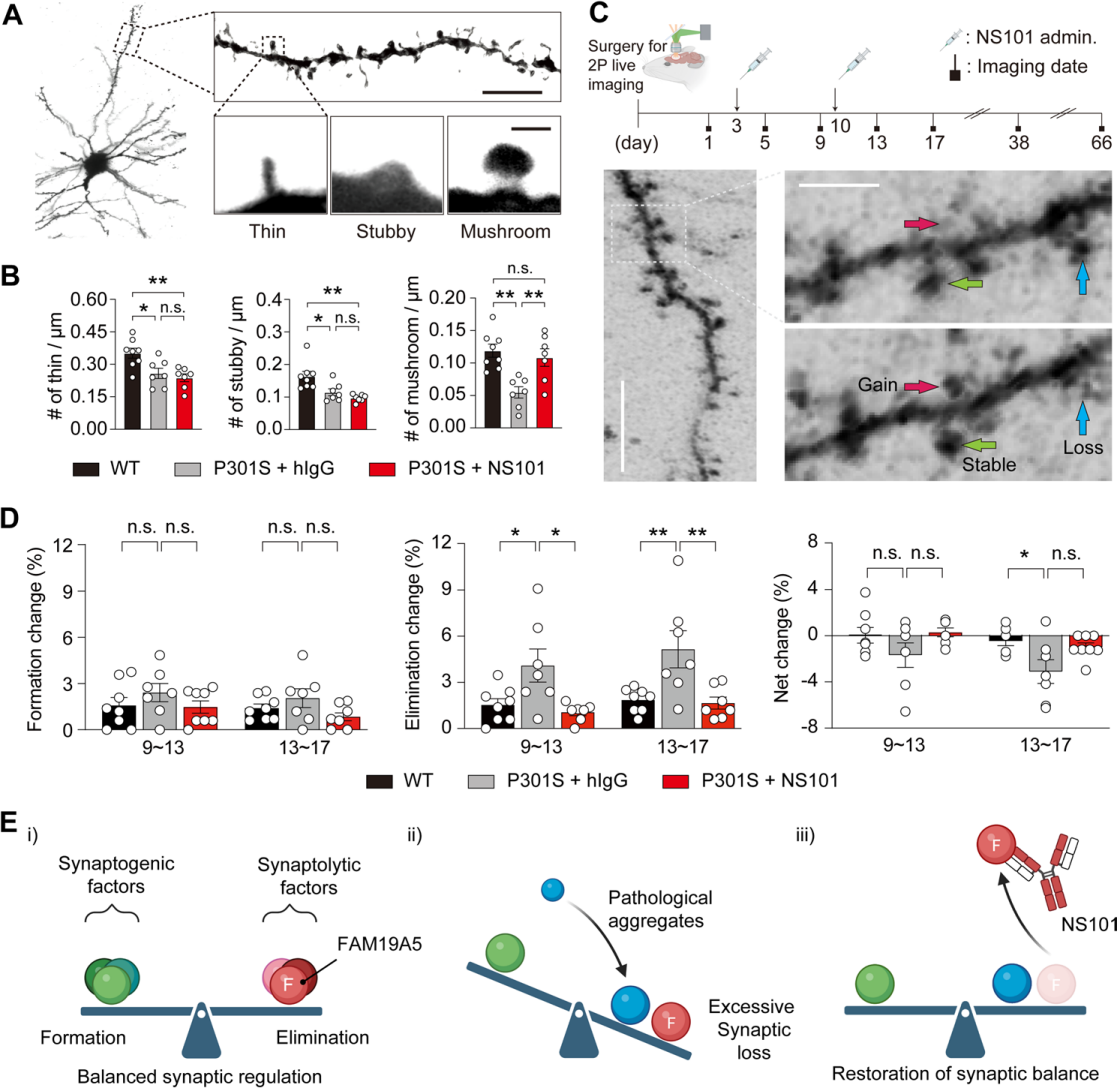
NS101 prevents synapse elimination, increasing the number of functional synapses in the P301S mice. (**A**) Representative Golgi staining image of a cortical neuron in the P301S mouse brain for spine classification. Scale bar, 10 μm (dendrite), 1 μm (spine). (**B**) Quantification of the number of different types of spines before and after NS101 treatment (*n* = 8 to 7 per group). Data are presented as the mean ± SEM. One-way ANOVA followed by Tukey’s multiple comparison test was used to calculate P values (thin: F_2, 19_ = 7.840, *P* < 0.005; stubby: F_2, 19_ = 9.721, *P* < 0.005; mushroom: F_2, 19_ = 9.914, *P* < 0.005). n.s., not significant; *P < 0.05, **P < 0.01. Number of thin / µm; *P* = 0.0186 (WT vs. P301S + hIgG), *P* = 0.0044 (WT vs. P301S + NS101). Number of stubby / µm; *P* = 0.0151 (WT vs. P301S + hIgG), *P* = 0.0013 (WT vs. P301S + NS101). Number of mushroom / µm; *P* = 0.0013 (WT vs. P301S + hIgG), *P* = 0.0079 (P301S + hIgG vs. P301S + NS101). (**C**) Representative image of spine dynamics in the P301S mouse brain. After cranial window surgery, the number of dendritic spines in the somatosensory cortex was measured at the indicated time points. Gained, stable, and lost spines are indicated by red, green, and blue arrows, respectively. Scale bar, 10 μm (dendrite), 1 μm (spine). (**D**) Quantification of spine dynamics at days 9 ~ 13 and 13 ~ 17 in the P301S mice. The formation and elimination rates and their net changes after the intraperitoneal administration of 30 mg/kg NS101 (*n* = 7–8 per group). Data are presented as the mean ± SEM. One-way ANOVA with Bonferroni’s multiple comparisons test was used to calculate P values (Formation change: F_5, 40_ = 1.444, *P* = 0.2298; Elimination change: F_5, 38_ = 5.618, *P* < 0.001; Net change: F_5, 39_ = 3.169, *P* < 0.05). n.s., not significant; *P < 0.05, **P < 0.01. Elimination change; *P* = 0.0463 (Day 9 ~ 13, WT vs. P301S + hIgG), *P* = 0.0166 (Day 9 ~ 13, P301S + hIgG vs. P301S + NS101), *P* = 0.0059 (Day 13 ~ 17, WT vs. P301S + hIgG), *P* = 0.0046 (Day 13 ~ 17, P301S + hIgG vs. P301S + NS101). Net change; *P* = 0.0398 (Day 9 ~ 13, WT vs. P301S + hIgG). (**E**) Illustration of synaptic balance restored by NS101. (i) Synaptic balance depends on the interplay between synaptogenic and synaptolytic factors for proper synapse formation and elimination. (ii) Aggregate toxicity can disrupt this balance, causing excessive synapse loss. (iii) NS101 targets FAM19A5 to reduce excessive elimination, restoring equilibrium and promoting synapse recovery

The correlation between the FAM19A5 and pTau proteins raises a hypothesis that synaptic balance, precisely regulated by synaptolytic factors including FAM19A5, is disrupted by the accumulation of pTau aggregates, leading to excessive synapse loss. By inhibiting FAM19A5 with NS101, we aimed to restore this balance by mitigating FAM19A5-mediated synapse elimination. To investigate this possibility, 6-month-old P301S mice, a model of advanced AD exhibiting progressive synapse loss in the cortex due to the accumulation of pTau proteins [47, 48, 49], were IV administered 10 mg/kg NS101 weekly for 4 weeks. P301S mice showed a notable reduction in spine density in the prefrontal cortex compared to that of their WT littermates. In particular, there was a marked reduction in the number of mushroom spines, which are mature spines capable of signal transmission. However, after NS101 administration, the mushroom spine density rebounded, reaching the level observed in WT mice (Fig. 5A and B). Meanwhile, the densities of other spine types remained relatively unaffected by NS101 treatment. To further explore potential sex differences in NS101’s efficacy, each type of dendritic spine was analyzed by sex. No significant sex-based differences were observed in spine subtype distribution within each treatment group. Notably, although statistical significance was primarily detected in male P301S mice, NS101 treatment consistently showed a trend toward increased mushroom spine density in both male and female P301S mice, while stubby and thin spine densities remained unaffected (Supplementary Fig. 12). These findings suggest that NS101 may selectively restore mushroom spines across sexes, despite current sample size limitations.

To investigate whether there was recovery in the balance of synapse formation and elimination after NS101 administration, we employed two-photon live imaging to trace spine changes in the somatosensory cortex of 7-month-old P301S mice following NS101 treatment for 66 days (Fig. 5C). Notably, under physiological conditions, only a small fraction of spines are formed and eliminated at a similar rate [50]. Consequently, the net balance of spine formation and elimination reaches a steady state of zero, maintaining a stable state [51]. Compared to WT mice, P301S mice exhibited a significantly greater rate of spine elimination, while spine formation rates remained unchanged between the groups. Consequently, the net change decreased significantly from 0% to -3.5%. Following two systemic injections of NS101 at weekly intervals, the spine elimination rate in P301S mice significantly decreased, resulting in the equilibrium of a net change comparable to that of WT mice (Fig. 5D). The rebalancing effect on spine elimination persisted for up to 2 months but did not reach statistical significance (Supplementary Fig. 13).

These results support the hypothesis that NS101 can restore the balance between synapse formation and elimination. Synaptic elimination, properly regulated by synaptolytic factors including FAM19A5, is exacerbated by pathological aggregates such as Aβ plaques or Tau tangles in AD, leading to excessive synapse loss [18]. By targeting FAM19A5 rather than pathological aggregates, NS101 restores the normal balance between the synapse formation and elimination. The sustained synapse formation and reduced synaptic elimination rate observed in this study can contribute to the preservation of synapses and the subsequent increase in stable and functional spines such as mushroom spine (Fig. 5E).

### NS101 restores synaptic activity and cognitive behaviour in mouse models of AD

**Fig. 6:**
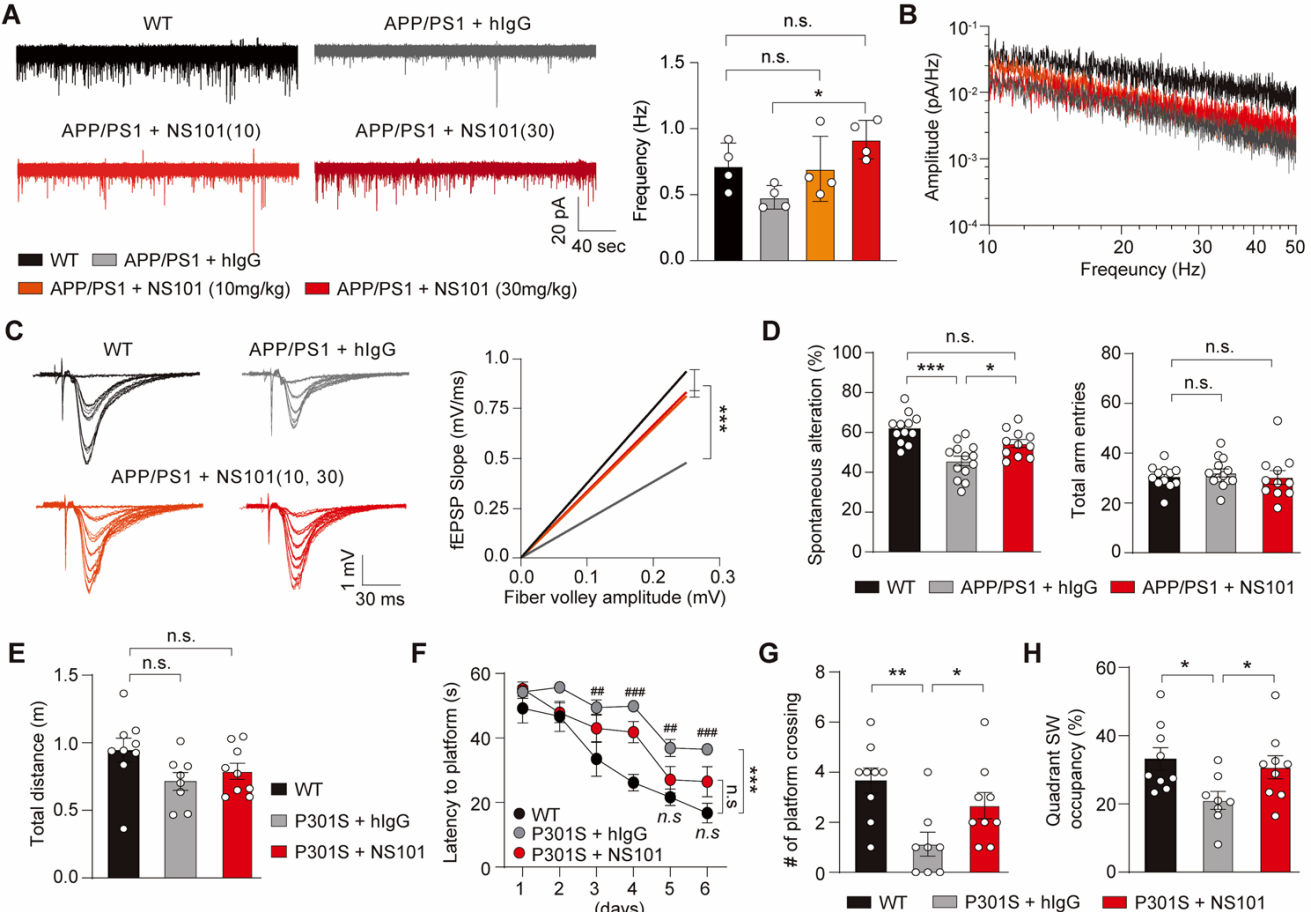
NS101 restores synaptic activity and cognitive behaviour in mouse models of AD. (**A**) Representative mEPSC traces in the hippocampal CA1 neurons of APP/PS1 mice and their frequency quantification (*n* = 4 per group). Data are presented as the mean ± SEM. One-way ANOVA followed by Tukey’s multiple comparison test was used to calculate P values (F_3, 12_ = 4.239, *P* < 0.05). n.s., not significant; *P < 0.05 (*P* = 0.0178, APP/PS1 + hIgG vs. APP/PS1 + NS101). (**B**) Power spectrum analysis, displaying the average line of the dominating spikes before and after NS101 administration. (**C**) Representative input‒output traces and fEPSP slopes plotted against fiber volley amplitudes in the hippocampal CA1 neurons of WT and APP/PS1 mice treated with either hIgG or 10 mg/kg or 30 mg/kg NS101 (*n* = 5 per group). One-way ANOVA followed by Tukey’s multiple comparison test was used to calculate P values (F_3, 800_ = 38.27, *P* < 0.0001). (**D**) Quantification of short-term memory in APP/PS1 mice using the Y-maze test (*n* = 11–13 per group). One-way ANOVA followed by Tukey’s multiple comparison test was used to calculate P values (Spontaneous alteration: F_2, 33_ = 13.85, *P* < 0.0001; Total arm entries: F_2, 33_ = 0.2737, *P* = 0.7623). Spontaneous alteration; ***P < 0.001 (WT vs. APP/PS1 + hIgG), *P < 0.05 (*P* = 0.0253, APP/PS1 + hIgG vs. APP/PS1 + NS101). Evaluation of spatial learning and memory via the Morris water maze as determined by (**E**) total distance travelled, (**F**) latency to reach the platform, (**G**) number of platform crossings, and (**H**) quadrant occupancy (*n* = 9 to 12 per group). Data are presented as the mean ± SEM. Two-way ANOVA followed by the Newman‒Keuls multiple comparisons test (for F) was used to calculate P values (F_2, 168_ = 34.15, *P* < 0.0001). ANOVA between groups comparing to WT; n.s., not significant; *P < 0.05, **P < 0.01, ***P < 0.001. Newman‒Keuls multiple comparisons test comparing to WT (E: F_2, 23_ = 2.608, *P* = 0.0953; G: F_2, 23_ = 6.236, *P* < 0.01; H: F_2, 23_ = 3.996, *P* < 0.05); *n.s.*, not significant; ^#^*P* < 0.05, ^##^*P* < 0.01, ^###^*P* < 0.001. *P* = 0.0010 (Day 3, WT vs. P301S + hIgG), *P* = 0.0017 (Day 5, WT vs. P301S + hIgG). Unpaired T test (for G-H) was used to calculate P values; *P < 0.05, **P < 0.01. Number of platform crossing; *P* = 0.0024 (WT vs. P301S + hIgG), *P* = 0.0498 (P301S + hIgG vs. P301S + NS101). Quadrant SW occupancy; *P* = 0.0121 (WT vs. P301S + hIgG), *P* = 0.0422 (P301S + hIgG vs. P301S + NS101)

Similar to P301S mice, APP/PS1 mice exhibit well-characterized synaptic deficits due to amyloid accumulation [52, 53, 54], making them a suitable model for studying synaptic pathology and its potential reversal. We investigated whether the increase in mature spine density associated with the restoration of synaptic balance translated into enhanced synaptic functions in AD. Thirteen-month-old APP/PS1 mice were IV administered NS101 weekly for 4 weeks. Subsequently, we measured the miniature excitatory postsynaptic currents (mEPSCs) in the CA1 pyramidal neurons of the hippocampus. The frequency of mEPSCs, which arise from the spontaneous release of neurotransmitters, was significantly lower in APP/PS1 mice than in their WT littermates. However, NS101 treatment restored the mEPSC frequency to levels comparable to those of WT littermates (Fig. 6A), consistent with the observations of increased intensities of synapse marker proteins in the hippocampus induced by NS101 (Supplementary Fig. 14). These findings, along with the slightly increased mEPSC amplitude (Fig. 6B), suggest that NS101 strengthens synaptic function. Synaptic strengthening by NS101 was further confirmed through field excitatory postsynaptic potential (fEPSP) measurements, revealing enhanced fEPSP slopes and fiber volley amplitudes (Fig. 6C).

The synaptic restoration directly correlated with significant improvements in cognitive performance. Disruption of excitatory synaptic architecture in the hippocampus especially in the CA1 subfield and dentate gyrus, and the medial prefrontal cortex is a well-established driver of memory impairment in AD, contributing to deficits in both short term and spatial working memory [55, 56]. In line with this, synaptic deterioration in APP/PS1 and P301S mice manifested behaviourally as decreased spontaneous alternation in the Y-maze and impaired spatial navigation in the Morris water maze. In the Y maze test, NS101 administration improved the spontaneous alternation rate of APP/PS1 mice to WT levels, while the total number of arm entries remained similar across all groups (Fig. 6D). Additionally, the cognitive improvement resulting from the neutralization of FAM19A5 was demonstrated in another AD mouse model, such as the 5XFAD [24]. We then conducted the Morris water maze test with P301S mice. All groups exhibited similar locomotion abilities (Fig. 6E). P301S mice took significantly longer to locate the platform than did WT mice. However, the NS101-treated P301S mice found the platform in a significantly shorter time (Fig. 6F). The performance of the NS101-treated P301S mice was similar to that of the WT mice in terms of quantitative indicators such as platform crossing and spatial preference (Fig. 6G and H). These results suggest that NS101 ameliorates the spatial learning and memory deficits associated with AD.

We investigated whether NS101-induced functional recovery in the AD brain is linked to the reduction of AD pathogens. NS101 significantly reduced pTau levels in the cerebral cortex but not in the hippocampus of P301S mice (Supplementary Fig. 15A-C). NS101 did not alter Aβ levels in either the cerebral cortex or hippocampus of APP/PS1 mice (Supplementary Fig. 15H-J). These findings suggest that restoring synaptic function can be achieved independently of Aβ reduction, as previously observed in other studies [35]. NS101 did not significantly alter microglial or astrocyte activation (Supplementary Fig. 15D-G, K-M). Overall, NS101 exerted minor effects on AD pathogens and glial activation but restored synaptic activity and cognitive function primarily by reestablishing synaptic balance through targeting FAM19A5.

## Discussion

This study identified FAM19A5 as a novel regulator of synaptic balance. Its strong binding to the FB domain of LRRC4B disrupted the interaction between LRRC4B and PTPRF, potentially triggering synapse disassembly. Overexpression of FAM19A5 in primary cultured neurons or treatment of neurons with FAM19A5 decreased the density of spine in contact with the presynaptic marker SYP. However, these synapse-reducing effects were reversed by NS101, which blocks the binding of FAM19A5 to LRRC4B. These results suggest that FAM19A5 functions as a negative regulator in synapse dynamics.

Synapses undergo dynamic formation and elimination processes throughout life, maintaining a delicate balance that serves as a crucial mechanism for regulating neural plasticity [57, 58]. This process is regulated by various synaptogenic factors and synaptolytic factors, including FAM19A5. However, in neurological diseases, pathological factors can disrupt this balance [59], leading to an excessive rate of synapse elimination and subsequent synaptic loss [19, 60]. This synaptic loss generally occurs early in the disease process [61], but even then, new synapses continue to form, albeit at a lower rate than the elimination rate. This persistent formation suggests the potential for reversibility of synaptic balance through reducing the elimination rate, offering hope for preventing or even reversing the disease process. Therefore, deepening our understanding of the mechanisms governing synapse dynamics may pave the way for more potent treatments for neurological diseases.

The tauopathy P301S mice showed lowered spine density compared to WT mice likely due to an increased spine elimination rate rather than a lowered spine formation rate. Treating P301S mice with NS101 to inhibit FAM19A5 normalized the spine elimination rate to that of WT mice, restoring the overall spine balance and leading to recovery of mature synapse numbers and function. NS101 exhibited comparable efficacy in restoring synaptic function in the hippocampus of APP/PS1 mice, a model of amyloidopathy. These findings suggest that inhibition of a single synaptolytic factor, FAM19A5, even in the presence of external synapse disrupting factors, such as amyloid or pTau aggregates, can normalize the synaptic balance, restoring lost synapses and regaining impaired cognition across tauopathy and amyloidopathy conditions. Considering that microglia actively contribute to spine pruning under various conditions, including drug administration [26] and neurodegenerative diseases [62], it is possible that inhibiting FAM19A5 with NS101 enhances spine stability by reducing microglia-mediated spine pruning.

In addition to external synapse disrupting factors, increased levels of FAM19A5 in neurons that are injured or surrounded by lesions can contribute to the progressive loss of synapses. Recent single-cell/nuclear RNA-seq analyses revealed increased *FAM19A5* transcript levels in AD brain neurons [5], especially in tau-tangle-bearing neurons [41]. These findings are related to our current study, which demonstrated that FAM19A5 levels in human CSF increase with age in association with total and pTau levels. Additionally, our earlier study showed increased transcript levels in a mouse model of traumatic brain injury [6]. Therefore, normalizing the synaptic balance by inhibiting the common synaptolytic factor FAM19A5 with NS101 offers a promising therapeutic approach for various neurological diseases characterized by excessive synapse loss arising from either external synaptolytic factors and/or increased FAM19A5 levels.

Synaptic adhesion molecules participate in regulating synapse numbers via molecular processes, including synapse formation, maturation, and continuous reorganization [57, 63]. The role of synaptic adhesion molecules extends beyond just adhesive contacts between membranes, as they play a critical role in organizing synapses and modulating their functional integrity [64]. In particular, LRRC4B can form excitatory synapses via transsynaptic interactions with presynaptic protein tyrosine phosphatase receptors (PTPR-F, -D, and -S) [34, 65, 66]. Additionally, via its PDZ-binding domain, it binds to the postsynaptic scaffolding protein PSD95 and promotes clustering of NMDA and AMPAR receptors at the postsynaptic site [33, 34]. Thus, LRRC4B plays a crucial role in various stages of excitatory synapse formation and function, such as inducing the initial transsynaptic connection between axons and dendrites, clustering postsynaptic proteins, and controlling synaptic plasticity [32, 34]. Its widespread expression across various types of neurons in mice and humans has been revealed by recent single-cell RNA-seq [4, 67], further underscoring its role in general synaptic dynamics or balance.

The recent success of a synthetic synapse organizer in directly restoring functional synapses and improving cognitive function in a mouse model of AD underscores the critical role of synapse restoration for AD treatment [35]. This suggests that even under adverse conditions, enhancing synaptic structure can normalize transmission, potentially triggering a positive feedback loop between healthy neurons and their environment. Notably, both this study and our own research offer structure-guided molecular tools as promising avenues for normalizing synapse function in patients with neurodegenerative diseases. An important advantage of our work over the synthetic synaptic organizer is the noninvasive delivery of NS101 into the brain. Systemic administration of NS101 crossed the BBB and facilitated the transport of FAM19A5 from the brain to peripheral blood in mice, rats, and humans in a clinical trial. This consistency of effect across species is likely due to the identical sequence of the FAM19A5 protein in these species.

The transport of target molecules from the brain to peripheral blood via an antibody-mediated approach was recently demonstrated in another study in which an anti-Tau antibody captured brain Tau proteins and transported them to the periphery in both transgenic mice and humans [68]. In this study, the transfer of target molecules from the brain to peripheral blood was readily apparent, as the baseline levels of the target molecules were much greater in the brain than in the periphery. These findings, along with our results, suggest that antibodies can be effective tools for removing target molecules in the brain, potentially offering a treatment approach for various neurological diseases. Given that a single IV administration of NS101 induces the transport of brain FAM19A5 to the blood over a month, antibody therapy has a clear advantage in clinical studies over other biologics that require direct administration to the brain and have a short half-life in the blood [35, 69].

Although we demonstrated that the FAM19A5-LRRC4B complex is involved in synapse formation, several important questions remain to be addressed in future investigations. It is currently unclear whether FAM19A5 interacts with proteins beyond LRRC4B and its paralogs, raising the possibility that additional binding partners may contribute to its synaptic effects. Moreover, the precise molecular components that regulate the formation and dissociation of the FAM19A5-LRRC4B complex are not yet defined. In addition, the intracellular signaling pathways activated or inhibited by this complex remain to be elucidated. A deeper understanding of these molecular mechanisms would not only clarify the broader role of FAM19A5 in the synapse but also potentially reveal novel therapeutic targets for enhancing synaptic restoration in neurodegenerative diseases. In addition, further studies are needed to comprehensively evaluate potential sex-dependent effects of NS101, as all in vivo functional studies were conducted in male mice, with the exception of the Golgi staining analysis.

## Conclusions

Our study unravels the critical role of FAM19A5 in regulating synaptic balance by binding to the FB domain of LRRC4B, promoting the elimination of synapses. This finding suggests the therapeutic potential for strategies that disrupt this interaction. Notably, both the anti-FAM19A5 antibody NS101 and the engineered protein/peptide containing the FB domain successfully blocked FAM19A5 binding to LRRC4B, highlighting promising avenues for restoring synaptic balance. These approaches hold particular relevance for neurological diseases suffering from progressive loss of synapses, which are mainly controlled by the FAM19A5-LRRC4B interaction.

## Electronic supplementary material

Below is the link to the electronic supplementary material.

Supplementary Material 1

Supplementary Material 2

## Data Availability

The raw data are available upon request from the authors. All other data generated or analyzed during this study are provided in this published article and its supplementary information files.

## Abbreviations

AD: Alzheimer’s disease
Aβ: Amyloid beta
FAM19A5: TAFA chemokine like family member 5
LRRC4B: Leucine-rich repeat-containing protein 4B
IV: Intravenous
PTPRF: Protein tyrosine phosphatase receptor type F
pTau: Phospho-Tau
CSF: Cerebrospinal fluid
Co-IP: Co-immunoprecipitation
PSD95: Postsynaptic density protein 95
SYP: Synaptophysin
aa: Amino acid
SPR: Surface plasmon resonance
K_D_: Dissociation constant
WT: Wild-type
MT: Mutant
AUC: Area under the curve
BBB: Blood-brain barrier
median TE_max_: Median time to maximum effect
mEPSC: Miniature excitatory postsynaptic current
fEPSP: Field excitatory postsynaptic potential
