# Supplementary material for: Inhibition of FAM19A5 reverses synaptic loss and cognitive decline in mouse models of Alzheimer’s disease
Source: Alzheimers Res Ther. 2025 Jul 21;17:168. doi: 10.1186/s13195-025-01813-8 (PMC12281766; doi:10.1186/s13195-025-01813-8)

**Supplemental Figure of Original Western Blots**

Figure 1C


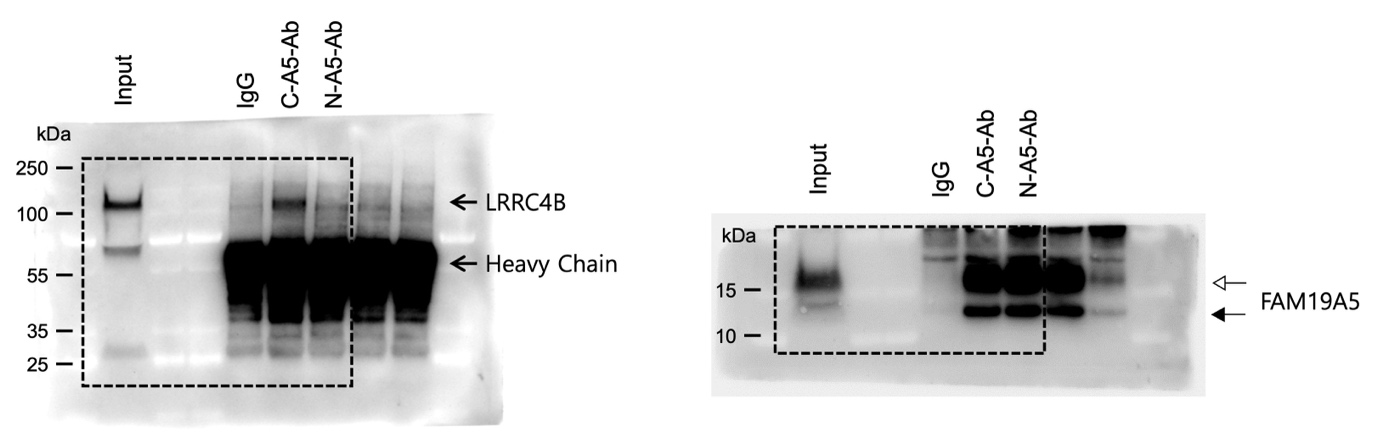


Figure 1D


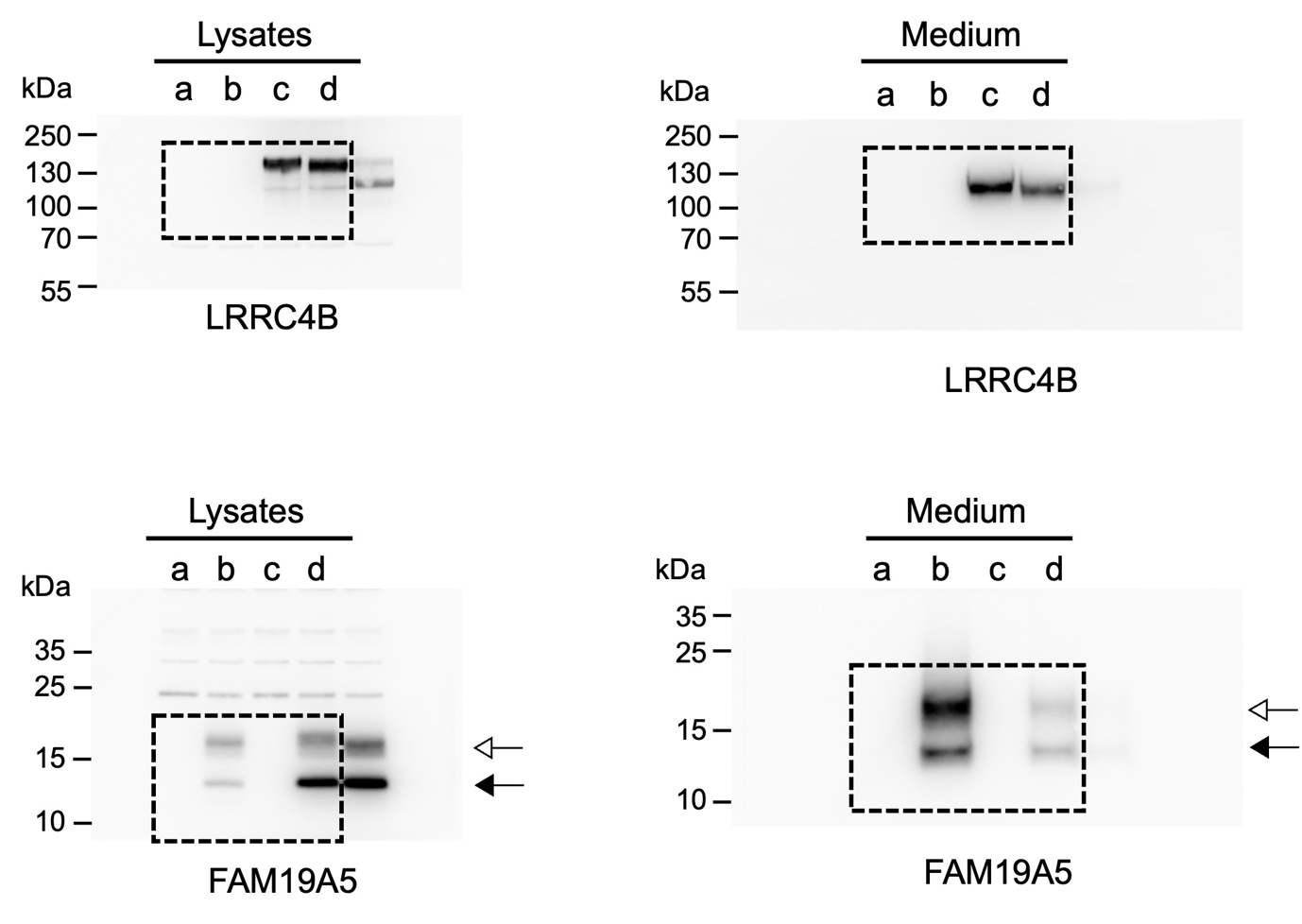


Figure 1E


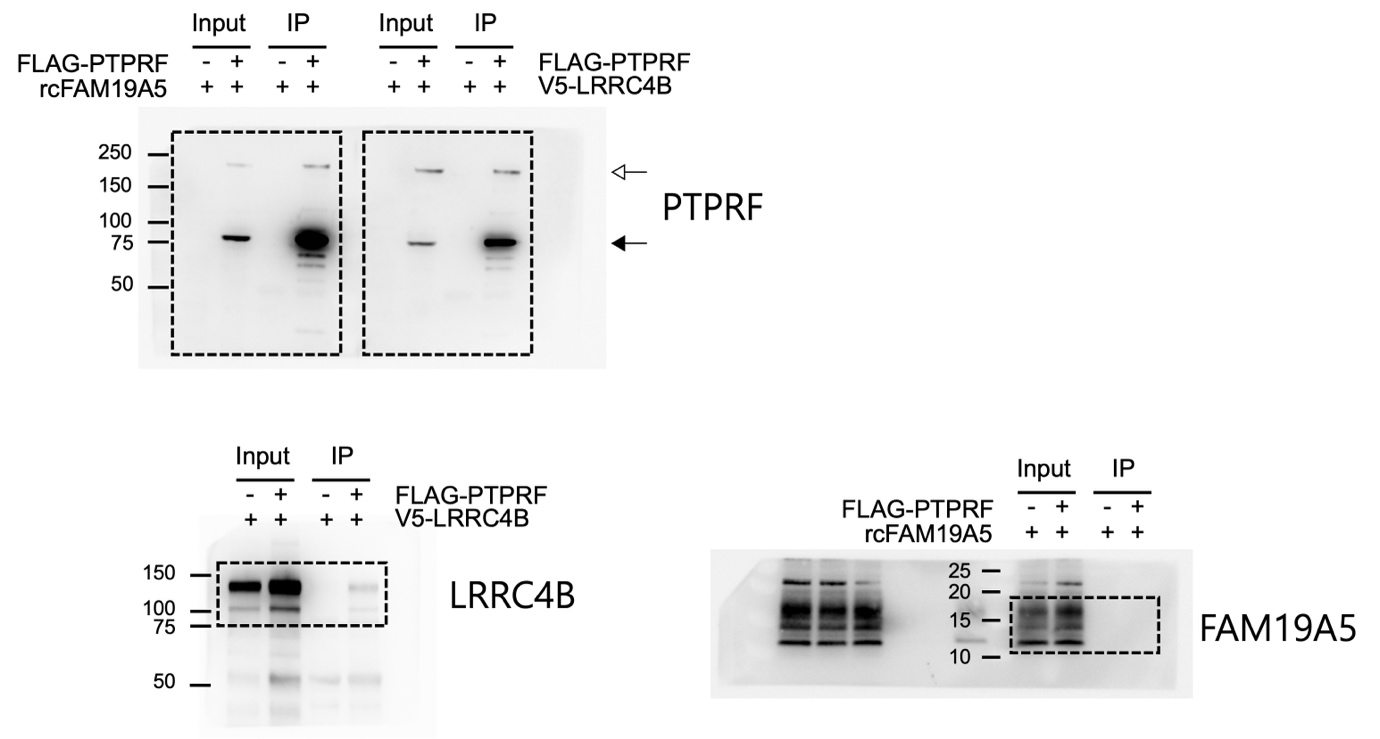


Figure 1H


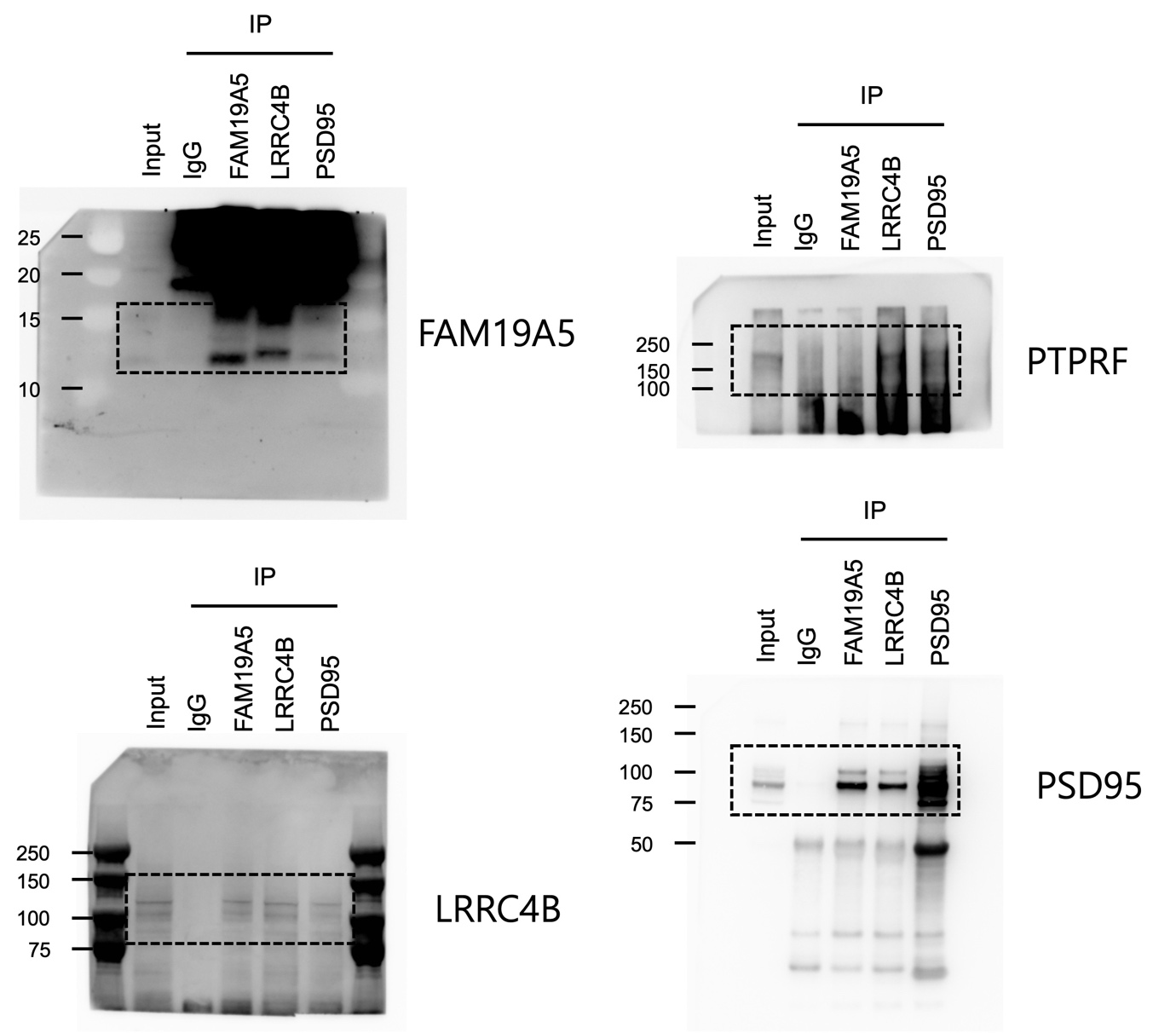


Figure 2B


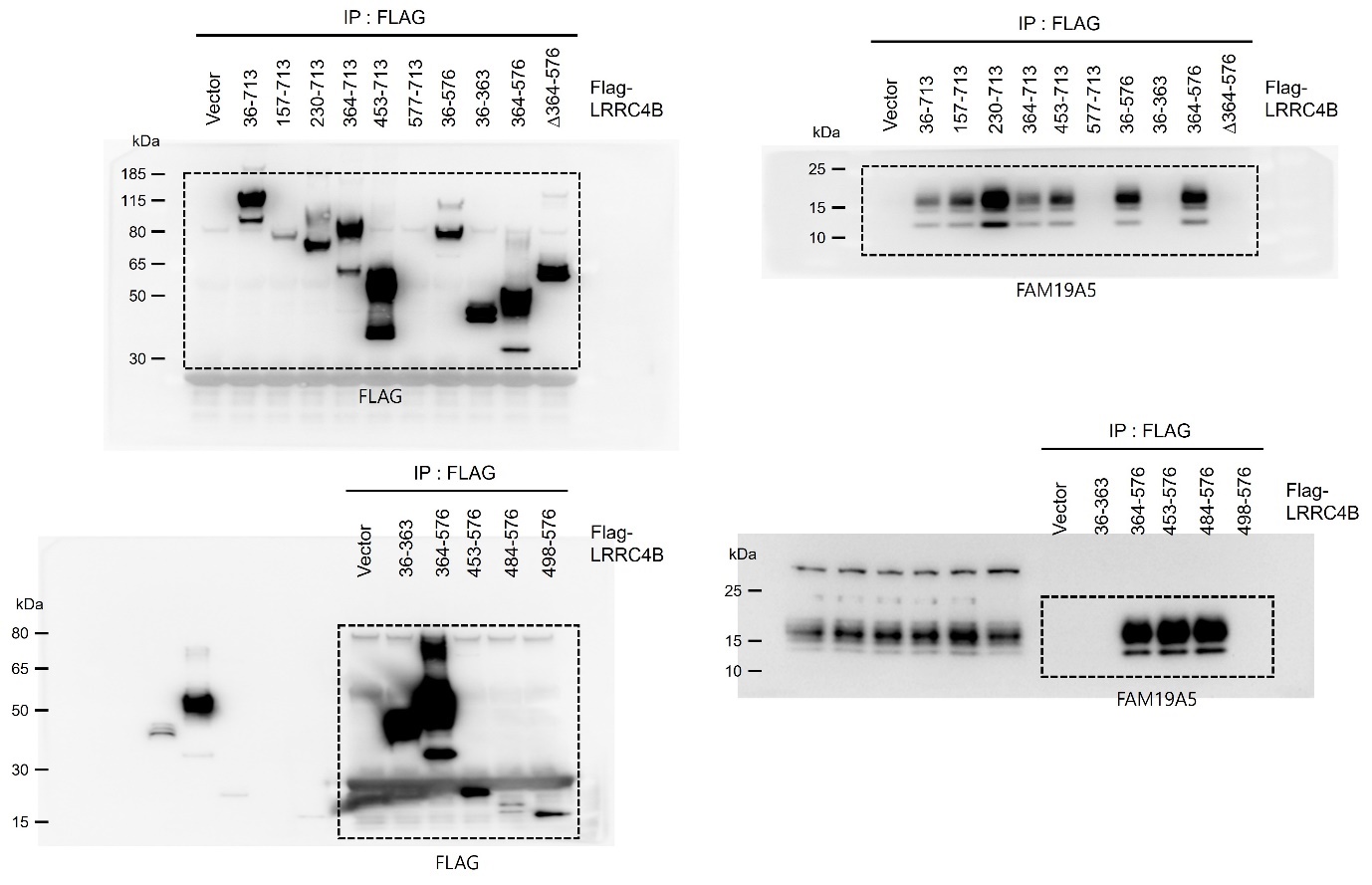


Supplementary Figure 2


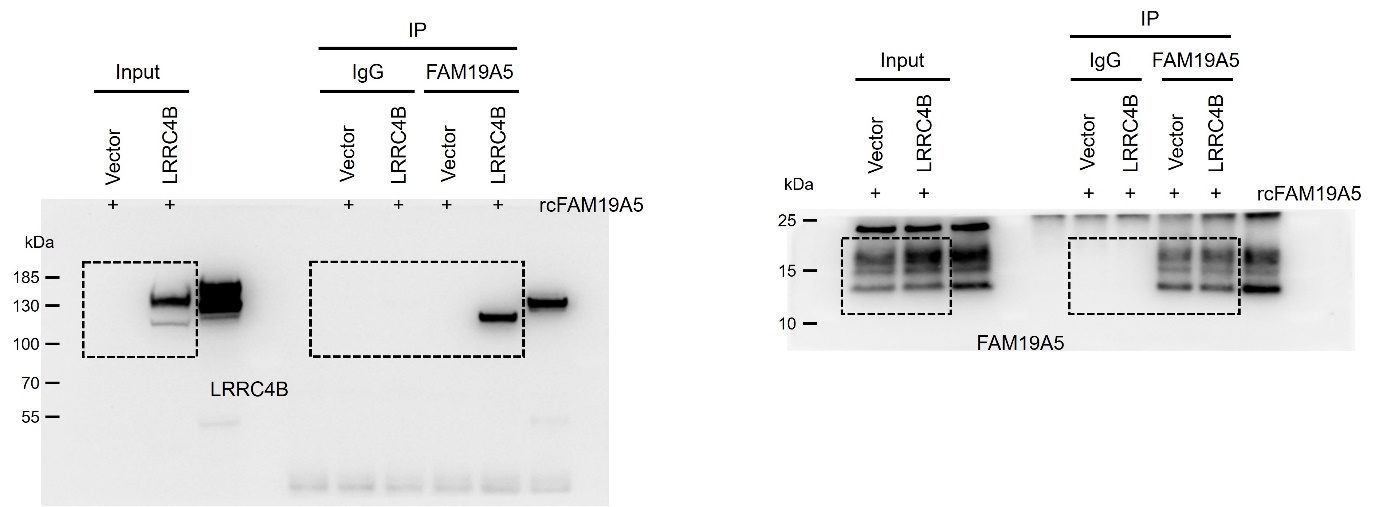


Supplementary Figure 5A


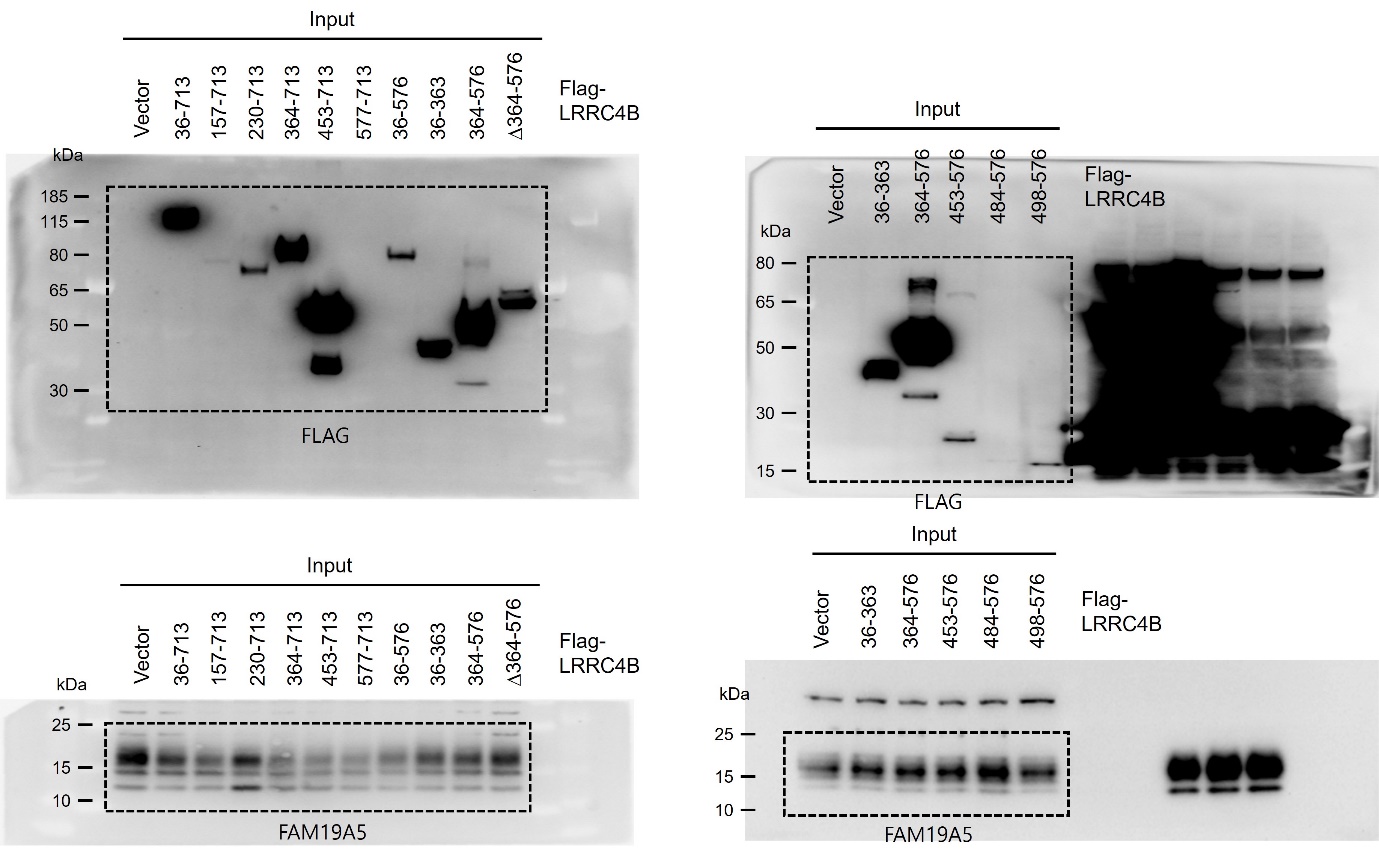


Supplementary Figure 7


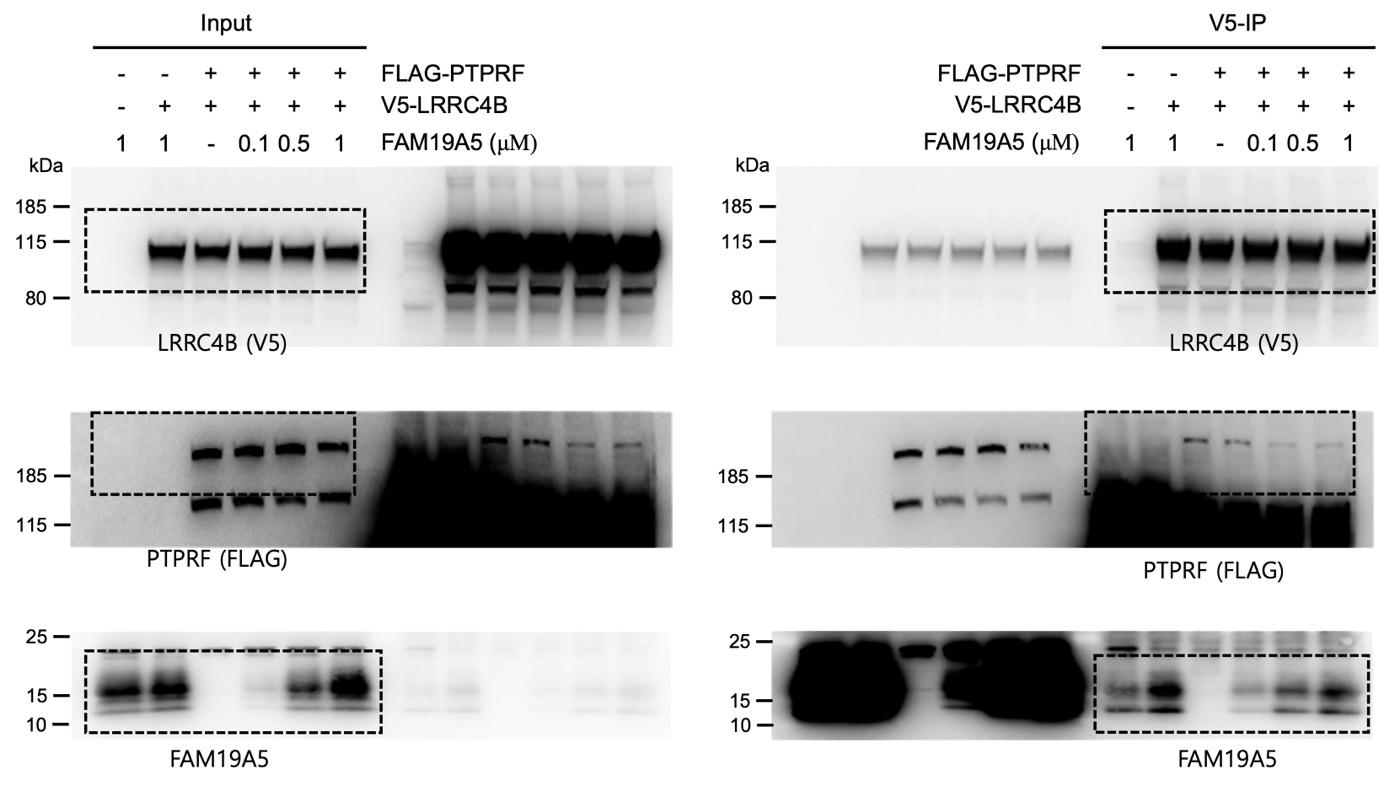


Supplementary Figure 8A


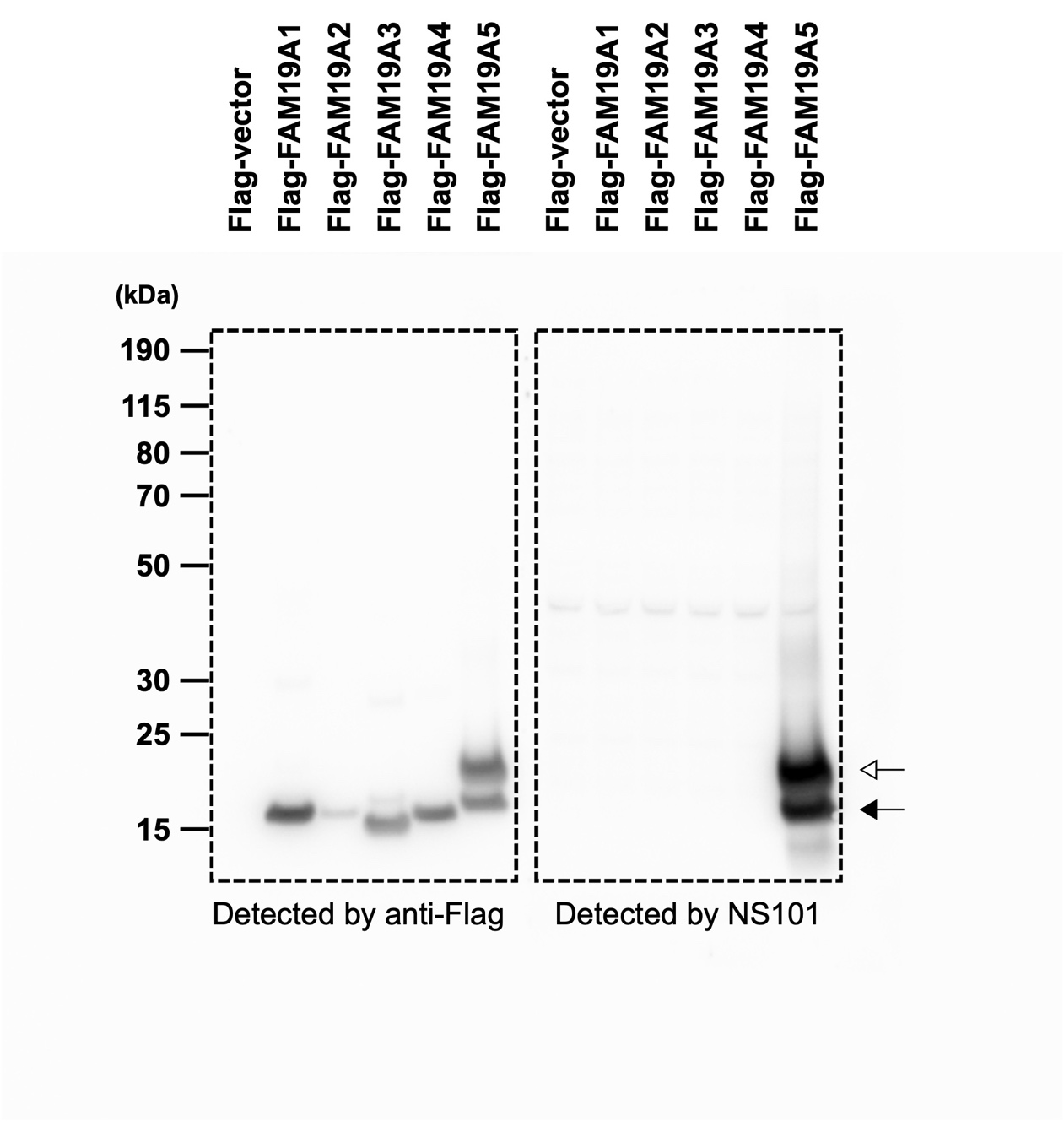


Supplementary Figure 11C


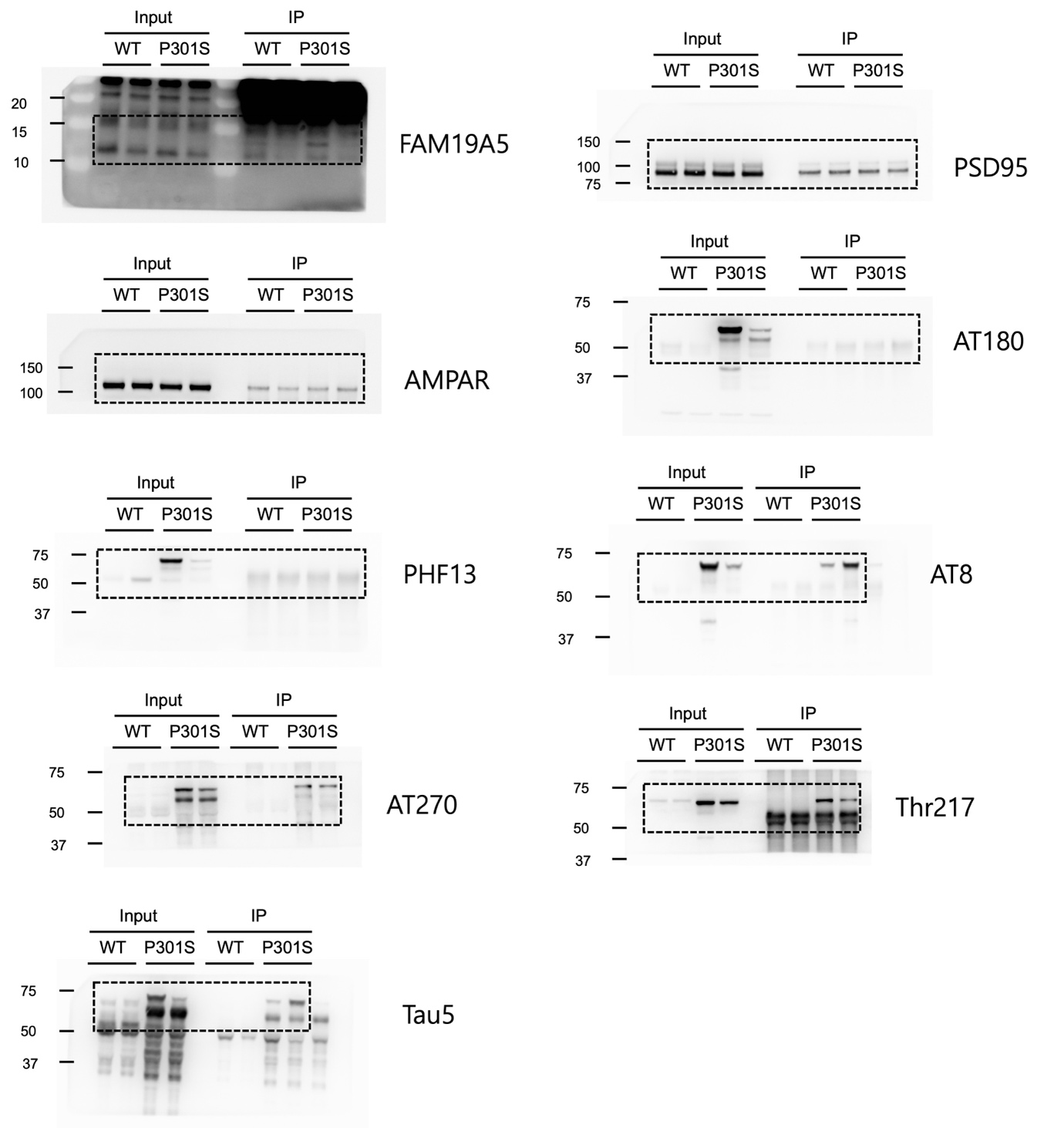

Supplement: Supplementary file 1 — Supplementary Material 1 [file 13195_2025_1813_MOESM1_ESM.docx]
