# Supplementary material for: Inhibition of FAM19A5 reverses synaptic loss and cognitive decline in mouse models of Alzheimer’s disease
Source: Alzheimers Res Ther. 2025 Jul 21;17:168. doi: 10.1186/s13195-025-01813-8 (PMC12281766; doi:10.1186/s13195-025-01813-8)
Supplement: Supplementary file 2 — Supplementary Material 2 [file 13195_2025_1813_MOESM2_ESM.docx]

**Supplementary Materials**

**Extended Methods**

**Cell lines**

HEK293 cells (ATCC# CRL-1573) were cultured in a humidified incubator at 37°C with 5% CO_2_. HEK293 cells were propagated in MEM with GlutaMAX^TM^ (Gibco) supplemented with 10% fetal bovine serum (Gibco) and 100 U/ml penicillin‒streptomycin (Gibco). For subculture, the cells were washed with DPBS (Gibco) and dissociated in 0.25% trypsin-EDTA (Gibco) for 5 min at 37°C.

**DNA constructs**

The pCMV20-Igκ_SP-FLAG vector was constructed by inserting a human Igκ signal peptide (SP) sequence in front of the FLAG tag of the pCMV20 plasmid (Sigma-Aldrich). Full-length (36-713) and serial deletion constructs (36-363, 36-576, 157-713, 230-713, 364-713, 453-713, 577-713, 364-576, *364-576, 453-576, 484-576 and 498-576) of human LRRC4B (NM_001080457.1) were subcloned and inserted into the pCMV20-Igκ SP-FLAG vector. The pCMV20-Igκ SP-hFc vector contained Igκ SP at the N-terminus of the multiple cloning site (MCS) and human-Fc (hFc) at the C-terminus in the pCMV20 plasmid. The ectodomain (36-576) and fragment (453-576) of human LRRC4B were inserted into the MCS of the pCMV20-Igκ_SP-hFc vector. The mutant constructs in which Thr488 and Thr489 of LRRC4B were substituted with Ala or Ser were subcloned and inserted into the pCMV20-Igκ_SP-FLAG or pCMV20-Igκ_SP-hFc vector. FAM19A5 (WT and MT) and LRRC4B fragments (36-576 and 453-576) were inserted into the pCAG1.1-Igκ_SP-His-TEV vector. The mutant constructs in which Arg58, Arg59, and/or Arg125, Lys127 of FAM19A5 were substituted with Ala were subcloned and inserted into the pCAG1.1-Igκ_SP-His-TEV vector. Full-length human LRRC4B was subcloned and inserted into the pcDNA6-V5-His vector (Invitrogen). FAM19A5 (NM_001252310.1) without tags was subcloned and inserted into the pcDNA3.1 vector (GenScript). The ectodomain (30-1263) of PTPRF (NM_002840.4) was inserted into the pCMV20-Igκ_SP-hFc vector. All cloning was performed using the AccuRapid™ Cloning Kit (Bioneer) according to the manufacturer’s instructions. The primer sets used for cloning are listed in Supplementary Table 1. Full-length human LRRC4B (OHu30422) and PTPRF (OHu02063) plasmid DNAs were purchased from GenScript.

**Purification of recombinant proteins**

Recombinant His-tagged proteins were purified according to methods described previously.^1^ In brief, FAM19A5 (WT and MT) and LRRC4B(36-576) with N-terminal 6xHis tags, followed by a tobacco etch virus protease (TEV) recognition sequence, were cloned and inserted into the pCAG1.1 vector (a gift from Jong-Ik Hwang) and expressed in Expi293F cells (Gibco, A14527). The supernatant containing His-TEV-FAM19A5 was collected for Ni-NTA affinity chromatography (Cytiva). After being washed with binding buffer containing 20 mM Tris-HCl (pH 7.5) and 200 mM NaCl, the proteins were eluted using binding buffers with increasing concentrations of imidazole (from 5 to 500 mM). Proteins eluted from 50 to 500 mM imidazole fractions were concentrated using Amicon® Ultra15 Centrifugal Filter Units (Millipore), and the buffer was exchanged for 1x DPBS (Gibco). The purified His-TEV-FAM19A5 proteins were digested overnight at 30°C with AcTEV protease (Invitrogen) to remove the His-tag and TEV sequence. The AcTEV protease containing the His-tag was then eliminated by overnight incubation with Ni-NTA, and digestion was confirmed using an anti-His tag antibody (Abcam). LRRC4B (ectodomain and fragments) and PTPRF (ectodomain) were cloned and inserted into the pCMV20-Igκ_SP-hFc vector and expressed in Expi 293F cells. The supernatant containing the hFc-fused proteins was collected using an rProtein A GraviTrap (Cytiva), and the hFc-fused proteins were eluted using an Ab buffer kit (Cytiva) following the manufacturer’s instructions.

**Production of anti-FAM19A5 antibodies**

We generated two chimeric chicken/human monoclonal antibodies against FAM19A5, named N-A5-Ab and C-A5-Ab, by immunizing chickens (Gallus gallus domesticus) with purified recombinant FAM19A5 using a previously described method.^1^ N-A5-Ab and C-A5-Ab were further deimmunized and optimized by amino acid substitution to generate NS101 and SS01, respectively.

**Production of protein fragments**

To elucidate the key residues in the FB domain of LRRC4B involved in FAM19A5 binding, we generated custom-made mutant peptides in which each single residue was substituted with Ala or Asn. The amino acid sequences are listed in Supplementary Table 2. To identify the epitopes of the FAM19A5 antibodies, the FAM19A5 linear sequence was divided into six groups labeled F1 to F6. Further analysis was conducted to determine the crucial residue for binding, in which each single residue in F2 was replaced with Ala. The relevant proteins and their amino acid sequences are listed in Supplementary Tables 3 and 4.

**Co-immunoprecipitation**

For human CSF Co-IP, pooled human CSFs (Innovative Research) were mixed with 0.5% NP40 and 50 μL of Pierce Protein A/G Plus Agarose (Thermo Scientific) that had been preincubated with 10 μg of anti-human IgG, N-A5-Ab, and C-A5-Ab. The mixtures were rotated at 4°C overnight and subsequently washed, after which the protein complexes were resolved by SDS‒PAGE.

For Co-IP of endogenous proteins in mice, the cerebral cortex was dissected and lysed in RIPA buffer supplemented with 25 mM Tris-HCl, 150 mM NaCl, 1% NP-40, 1% sodium deoxycholate, 0.1% SDS, and a protease and phosphatase inhibitor cocktail (Thermo Scientific). The tissue was then homogenized and subjected to sonication to ensure complete lysis. To remove insoluble material, the lysate was centrifuged at 16,000 × g for 30 min at 4°C. Subsequently, 1 mg of cortex lysate supernatant was incubated with 30 μL of Protein G Dynabeads (Invitrogen) preincubated with the appropriate antibodies (the information for the antibodies is in Supplementary Table 5): anti-FAM19A5 (S-A5-Ab), anti-LRRC4B (Alomone labs), and anti-postsynaptic density protein-95 (PSD95) (Invitrogen). The subsequent steps were conducted as described for the Co-IP procedure performed with HEK293 cells.

For Co-IP of synaptosomal fractions from P301S mice, synaptic proteins were isolated from the cerebral cortex and lysed in Syn-PER extraction buffer (Thermo Scientific) supplemented with a protease and phosphatase inhibitor cocktail (Thermo Scientific) according to the manufacturer’s protocol. Briefly, the cortical tissue was homogenized, and the homogenate was centrifuged at 1,200 × g for 10 min at 4°C to remove debris. The resulting supernatant was further centrifuged at 15,000 × g for 20 min at 4°C to separate the cytosolic fraction (supernatant) from the synaptosome pellet. The synaptosome pellet was then resuspended in Syn-PER buffer and kept on ice until further processing. For Co-IP, 0.3 mg of the synaptosomal fraction was incubated with 30 μL of Protein G Dynabeads (Invitrogen) preincubated with an anti-FAM19A5 antibody (S-A5-Ab). Subsequent steps were carried out according to the Co-IP protocol established for HEK293 cells.

**ELISA**

For the measurement of FAM19A5 in biofluids and tissues, we coated 96-well microplates with LRRC4B(453-576) protein or S-A5-Ab, which was diluted in 50 mM carbonate buffer (pH 9.6) to achieve a final concentration of 1 µg/ml. The plates were sealed and incubated overnight at 4°C. The following day, we washed the plates twice with 300 µL of washing buffer (PBS with 0.05% Tween 20) per well using a microplate washer (Tecan) and gently tapped them on a paper towel to remove any remaining solution. Subsequently, 200 μL of blocking buffer was added to each well, and the plates were sealed and incubated at 37°C for 1 hour. After two additional washes, 100 μL of both the standard solution and the samples were added to each well. The plates were sealed and incubated at room temperature for 90 min. Following another five washes, 100 μL of HRP-conjugated C-A5-Ab diluted in blocking buffer (PBS with 1% BSA and 0.05% Tween 20) to a final concentration of 0.2 μg/ml was added to each well and incubated at 37°C for 1 hour. Then, 100 μL of TMB solution (Thermo Scientific) was added to each well, and the plates were incubated at room temperature for 20 min. To stop the colorimetric reaction, 100 μL of 1 N sulfuric acid was used, and the optical density (OD) of each well was determined using a microplate reader (Molecular Devices) at 450 nm.

To measure CSF NS101, we used a pair of rabbit anti-human IgG heavy chain antibodies (Invitrogen) and HRP-conjugated goat α-human IgG kappa light chain antibodies (Thermo Scientific).

To assess the binding affinity between FAM19A5 and the LRRC4 family, we utilized various LRRC4/4B/4C deletion proteins, including LRRC4(39-527), LRRC4B(36-576), LRRC4B(453-576), LRRC4B(484-576), LRRC4B(498-576), and LRRC4C(45-527), as capture agents. We used both the WT and MT FAM19A5 proteins, which included FAM19A5(R58A, R59A), FAM19A5(R125A, K127A), and FAM19A5(R58A, R59A, R125A, K127A), for binding analysis. Detection was carried out using HRP-conjugated C-A5-Ab.

To examine the inhibition of FAM19A5-LRRC4B binding, we first coated plates with the LRRC4B(453-576) protein. Subsequently, we added the test sample and the FAM19A5 protein. The degree of binding inhibition was determined by detecting FAM19A5 using an HPR-conjugated C-A5-Ab. For the PTPRF-LRRC4B binding inhibition test, the LRRC4B(36-576) protein was coated on the plate, followed by the addition of the test sample and PTPRF protein. Binding inhibition was calculated by detecting PTPRF-hFc using an anti-human IgG Fc antibody.

The amino acid sequences used to elucidate the key residues in the FB domain of LRRC4B and to identify the epitopes of the FAM19A5 antibodies are listed in Supplementary Tables 3, 4, and 5.

**Immunoblots**

The cell lysates were prepared in lysis buffer containing 20 mM Tris-HCl (pH 7.5), 150 mM NaCl, 0.5% NP-40, and protease inhibitor cocktail (Thermo). The primary antibodies used are listed in Supplementary Table 5. Immunoblots of the cell lysates were performed as described previously^1^.

**Phase 1 trial of NS101**

The study NCT05143463 is a phase 1, double-blind, randomized, placebo-controlled, first-in-human (FIH) clinical trial designed to evaluate safety and tolerability, as well as pharmacokinetic (PK), pharmacodynamic (PD), and immunogenicity profiles of the study NS101 in healthy subjects under fasting conditions. We selected a healthy male volunteer population for this study because healthy subjects without any concomitant diseases or medications are a homogenous population, which allows proper evaluation of the safety, tolerability, and PK of NS101 without confounding factors. We assessed subjects against the inclusion and exclusion criteria, which were described in our clinical report (NCT05143463), to determine whether they were eligible to participate in this study. All the subjects were provided with informed consent forms (ICFs) in their preferred language (either French or English) for review. Prior to initiation of the study procedures, the ICF was verbally reviewed with the subjects by qualified staff, allowing sufficient time for review of the information provided and to answer any questions the subjects had. The subjects were informed of any developments or changes to procedures that could influence their continued participation in the study. The subjects did not anticipate any direct benefits from participation in this research study, with the exception that they received a health evaluation. The participants in this study were compensated for their time; however, they were not offered any incentives. NS101 infusion doses of 0.25, 0.75, 1.5, 3.0, 6.0, 12.0, 24.0, and 48.0 mg/kg (cohorts 1 to 8) were selected based on nonclinical pharmacology, PK, and toxicology studies in rats and monkeys. The study consisted of 8 cohorts, each including 8 subjects, randomized at a 3:1 ratio (6 subjects received NS101, and 2 received matching placebo), for a total of 64 subjects. For every cohort, the body weight of each subject measured the day before was used to calculate the exact individual dose required on a mg/kg basis based on their assigned dose level. The drug was administered on the morning of the day after the subjects had fasted overnight for at least 8 hours. The cohorts were dosed sequentially in an ascending fashion. The IV infusion was performed over a period of approximately 60 minutes in bed using aseptic techniques at a constant rate using a volume-controlled infusion device. At the end of the infusion, 3 mL of saline solution was injected to flush the remaining drug into the IV catheter. The end of the infusion was set to the end of the 3 ml flush.

In each cohort, a total of 21 blood samples were drawn into blood collection tubes (1 x 3 mL) containing plastic serum spray-coated silica before infusion and at 0.25 (±3 min), 0.5 (±3 min), 0.75 (±3 min), 1 (±3 min), 1.25 (±3 min), 1.5 (±3 min), 2 (±3 min), 4 (±3 min), 6 (±3 min), 8 (±3 min), 12 (±15 min), 24 (±15 min) (Day 2), 36 (±15 min) (Day 2), 48 (±15 min) (Day 3), 96 (Day 5±1), 168 (Day 8±1), 336 (Day 15±1), 504 (Day 22±2), 672 (Day 29±2), and 1416 (Day 60±3) hours after the start of infusion. Blood samples were kept at room temperature and were centrifuged at 1300 ±20 g for at least 10 minutes at room temperature (no more than 180 minutes passed between the time of each blood draw and the start of centrifugation). Two aliquots of at least 0.5 mL (when possible) of serum were dispensed into polypropylene tubes as soon as possible. The aliquots were stored at -80°C until ELISA.

Subjects assigned to Cohorts 5 through 8 were randomized for collection of a CSF sample at one of the following timepoints: 24 (±3 horus), 36 (±3 horus), 168 (day 8±1), or 336 (day 15±1) hours poststart of infusion. Each of the 4 timepoints was assigned to 2 subjects. The sentinel subjects were randomized to the same timepoint to ensure that each timepoint was assigned to at least 1 subject receiving NS101. A single CSF sample was collected via lumbar puncture. The volume of collected CSF per sample did not exceed approximately 6 ml. A stabilizing agent (10% BSA in 1X PBS with 5% Tween-20) was added, and mixed with the NS101 CSF samples (no more than 8 minutes between sample collection and mixing with the stabilizing agent). Two aliquots of at least 0.5 mL (when possible) of CSF were dispensed into polypropylene cryovials as soon as possible. The aliquots were stored at -80°C (±10°C) until ELISA.

The clinical study protocol, any relevant associated documents, and ICFs were reviewed and approved by an Institutional Review Board (IRB, **#**00000971) prior to beginning the associated study procedures. The ethics committee that reviewed the study was Advarra, an independent service provider located in Aurora, Ontario, Canada. The IRB had no representatives from Syneos Health or Sponsor and was, therefore, completely independent. All clinical work was conducted in compliance with Good Clinical Practices (GCP) as referenced in the International Council for Harmonization (ICH) guidelines (ICH E6), Good Laboratory Practices (GLP) as referenced in the ICH guidelines, and all applicable regulations, including the Federal Food, Drug and Cosmetic Act, U.S. applicable Code of Federal Regulations (CFR) Title 21, and any IEC requirements relative to clinical studies.

**Surface plasmon resonance (SPR)**

SPR experiments were carried out on a Biacore 8K (Cytiva, for affinity between NS101 and FAM19A5) or a Biacore t200 (Cytiva, for affinity between FAM19A5 and LRRC4B) with the active temperature controlled at 25°C following the manufacturer’s protocols. The running buffer was 1 × HBS-EP (10 mM HEPES, 150 mM NaCl, 3 mM EDTA, 0.05% Tween 20, pH 7.4). For immobilization, 6xHis-LRRC4B(453–576) in 10 mM sodium acetate, pH 4.5 buffer equivalent to 0632–638 resonance units (RU), was injected onto a nitrilotriacetic acid (NTA) chip at a 30 µl/min flow rate. FAM19A5 at increasing concentrations (0.78, 1.56, 3.12, 6.25, and 12.5 nM) was diluted in running buffer and flowed across the immobilized 6xHis-LRRC4B(453–576) for 180 s at a flow rate of 30 μl/min (association). The sample was replaced with running buffer for 240 s (disassociation). The chip surface was regenerated with 350 mM EDTA and 500 mM HBS-P imidazole. For all the samples, the blank injection with buffer alone was subtracted from the resulting reaction surface data. The data were analyzed using Biacore 8K evaluation software (Cytiva).

**In silico modeling of FAM19A5-LRRC4B**

We used AlphaFold2 to model the structure of the FAM19A5-LRRC4B complex. Input paired multiple sequence alignments (paired MSAs) were generated following the input generation protocol of RoseTTAFold. hMSAs for the FAM19A5 protein and LRRC4B extracellular domain were generated by an iterative sequence search against the UniClust30 database^2^ using HHblits.^3^ To predict complex structures, we generated paired MSAs based on individual MSAs by pairing sequences from the same species. To provide pseudomultimer inputs to AlphaFold2, we used a gap insertion trick that was previously applied to RoseTTAFold. The final models were ranked by the predicted TM score (pTM score), and the best scored model was used for further study after structural relaxation using Rosetta. To identify the salt bridge, PyMOL (Schrödinger, LLC) was used to identify the charged residues in the FAM19A5-LRRC4B complex. The atomic bonds between oxygen and nitrogen within 2.5-4.0 Å were subsequently screened.

**In silico residue scanning of the FAM19A5-FB complex**

We used the residue scanning module (Schrodinger Bioluminate®) for in silico residue scanning (e.g., Ala scanning) and to calculate the perturbation of protein-protein binding affinity, which was defined as the change in binding free energy. To evaluate the potential impact of a mutation, we used ΔAffinity and ΔStability (solvated) values.

**Primary neuronal culture**

Primary cortical or hippocampal neurons were prepared from postnatal C57BL/6 pups (Nara Biotech) at postnatal day 1 following established procedures.^4^ Initially, the cortices were dissected in Hank’s buffered salt solution (HBSS) (Invitrogen) and then subjected to digestion with 2.5% trypsin for 15 minutes at 37°C. After this step, the supernatant was removed, and the tissues were washed with HBSS. The tissues were gently triturated, and the dissociated cells were plated on glass coverslips precoated with poly-D-lysine (in borate buffer at a concentration of 50 μg/ml, Sigma-Aldrich). These plated cells were housed in 60 mm culture dishes and cultured in minimum Eagle’s medium (MEM) supplemented with 0.5% glucose, 1 mM pyruvate, 1.2 mM L-glutamine, and 12% fetal bovine serum. After 6 hours of incubation, the medium was replaced with neurobasal medium (Invitrogen, Carlsbad, CA, USA) supplemented with 2% B-27 and 0.5 mM L-glutamine (Gibco). The cells were then maintained in a 5% CO_2_ humidified incubator at 37°C. Every 3–4 days, half of the medium in the culture dish was replaced with fresh culture medium.

For RNA-seq analysis, neurons were cultured for 16 days, and total RNA was isolated at various time points: 1, 3, 7, 10, and 16 days. The RNA was then subjected to RNA-seq analysis following established protocols.^5^

Primary cultured neurons were transfected using a calcium phosphate precipitation method. Briefly, 10 µg of plasmid DNA was mixed with 18 µL of 2M CaCl₂, and the final volume was adjusted to 150 µL with distilled water (DW). This solution was then combined with 150 µL of 2X HBS (Heptic Buffered Saline) and incubated at room temperature for 30 minutes. The mixture was subsequently applied to the primary neurons by adding dropwise to the culture medium.

**Immunocytochemistry and immunohistochemistry**

Immunocytochemistry for primary cultured neurons: For primary cultured neurons, fixation was carried out with 4% PFA, except for the spine density assay, which used a solution of 4% PFA. After three subsequent washes with ice-cold DPBS, the cells were blocked using buffer containing 3% bovine serum albumin (BSA) and 0.1% Triton X-100 in PBS for 30 minutes. The cells were then incubated with primary antibodies (anti-PTPRF (Santacruz), anti-SYP (Sigma) and anti-PSD95 (Invitrogen)), overnight at 4°C. After three additional washes with DPBS, the cells were incubated with secondary antibodies and 20 mM Hoechst solution (Invitrogen) and Phalloidin (Cytoskeleton) for 1 hour at room temperature. Following three final washes with DPBS, we captured fluorescence images using a confocal microscope (Leica), and the coverslips were mounted with mounting solution (Biomeda). All the images were processed using Las X software (Leica). For confocal microscopy, images were acquired using a Leica TCS SP8 confocal microscope with HC PL APO CORR CS2 40x/1.10 W and HC PL APO CS2 60×/0.40 objective (Leica). Images were acquired in 2D mode and Image resolution was set to either 2048 × 2048 or 4096 × 4096 pixels depending on magnification and zoom conditions. All the images were processed using Las X software (Leica). The quantification of synaptic markers and their colocalization was performed using ImageJ (NIH) with the colocalization plugin. The quantification of dendritic spine was performed from secondary dendrites.

Immunohistochemistry of brain slices: In the context of immunohistochemistry, the mice underwent transcardial perfusion with a solution of 4% PFA in PBS, and their isolated brains were subsequently postfixed in the same fixative for 24 hours. These brains were then cryoprotected in 30% sucrose, serially sectioned on a cryostat (40 𝛍m), and stored in a mixture of 50% glycerol and 50% PBS at −20°C until further use. The brain sections were blocked for 30 minutes in buffer composed of 3% BSA and 0.1% Triton X-100 in PBS. They were then incubated with primary antibodies diluted in blocking buffer overnight at 4°C. After this overnight incubation, the sections were subjected to three PBS washes and then incubated with secondary antibodies or Hoechst 33242 diluted in PBS for 30 minutes at room temperature. Subsequently, the sections were washed again, mounted, and observed using either a slide scanner (Zeiss) or a confocal microscope (Leica). For confocal microscopy, images were acquired using a Leica TCS SP8 confocal microscope with a HC PL APO CS2 20×/0.75 dry objective (Leica). Imaging was performed in 2D mode without z-stack acquisition, at a lateral resolution of 0.568 µm/pixel (x–y). The regions of interest (ROIs) within the CA1 pyramidal layer were manually selected for quantitative analysis. For slide scanner imaging, whole-slide fluorescent images were acquired using the Zeiss Axioscan Z1 slide scanner with a Plan-Apochromat 20x/0.8 M27 (Zeiss). Images were captured at a resolution of 0.325 µm/pixel (x–y). ROIs in the cerebral cortex and hippocampus were manually selected for analysis. Z-stacks were not acquired.

**Proximity ligation assay (PLA)**

A proximity ligation assay was employed to detect LRRC4B-FAM19A5 interactions within the neuronal environment following the manufacturer's protocol. Primary hippocampal neurons were fixed with 4% paraformaldehyde at 12 DIV. Following fixation, the cells were blocked with a solution containing 3% bovine serum albumin (BSA) in 0.1% Triton X-100 in phosphate-buffered saline (PBS) for 1 hour at room temperature. The primary antibodies used included the C-A5-Ab antibody, which was conjugated using the Duolink® In Situ Probemaker MINUS, anti-LRRC4B (Alomone Labs, #ANR-163), and anti-PSD95 (Invitrogen, #MA1-045) antibodies. These antibodies were added, and the samples were incubated overnight at 4°C. Following primary antibody incubation, secondary antibody (Donkey anti-mouse A650, Abcam, #ab96878) and phalloidin 488 were added. Duolink® In Situ PLA® Probe Anti-Rabbit PLUS was then applied for the secondary plus probe reaction, allowing for the formation of a circular DNA when in close proximity. The DNA circle was subsequently amplified via Duolink® In Situ Detection Reagents Red (DUO92008), and the resulting products were detected with complementary fluorescently labeled oligonucleotides. Confocal images were acquired using a Leica TCS SP8 confocal microscope equipped with the Lightning deconvolution module. Images were captured using a 63× oil or 40× water immersion objective lens. Image resolution was set to either 2048 × 2048 or 4096 × 4096 pixels depending on magnification and zoom conditions. All images were processed using the Leica Lightning adaptive deconvolution software to enhance resolution and reduce background signal.

**Golgi staining**

Golgi staining was performed according to the manufacturer's instructions (FD NeuroTechnologies, PK401). Whole fresh brains were collected and placed in impregnation solution. The solution was replaced after 24 h, and then the brains were incubated at room temperature in the dark for 2 weeks, with gentle shaking every 3 days to prevent precipitation of the impregnation chemicals. After the impregnation step, the brains were placed in a rehydration solution for 1 week. The brains were frozen in a container with dry-ice cooled isopentane (Sigma-Aldrich, M32631) and cryosectioned into 100 μm slices. Prefrontal cortex slices were mounted on gelatin-coated slides (FD NeuroTechnolgies, PO101). For the developing step, the slides were incubated for 10 min in staining solution. The slides were dehydrated with increasing concentrations of pure ethanol (Sigma-Aldrich, E7023), cleared with xylene (Daejung, 8587-4400), and then coverslipped with mounting medium (Sigma-Aldrich, 03989). High-resolution confocal images were acquired using a Leica TCS SP8 microscope with a 63×/1.40 oil-immersion objective and 3× digital zoom. Z-stack imaging was performed at 0.5 µm intervals across 160 optical sections, with an x–y resolution of 0.04 µm per pixel. Maximum intensity projection was applied for visualization and analysis.

**Hippocampal slice preparation and electrophysiology**

APP/PS1 mice and WT mice (13 months old) were anesthetized with isoflurane (5% isoflurane, 95% O_2_) and perfused with ice-cold sucrose artificial cerebrospinal fluid (aCSF) containing 195.5 mM sucrose, 2.5 mM KCl, 1 mM NaH2PO_4_, 32.5 mM NaHCO_3_, 11 mM glucose, 2 mM Na pyruvate, and 1 mM Na ascorbate (all chemicals from Sigma) bubbled with 95% O_2_/5% CO_2_ at a pH of 7.4. After perfusion, the brains were quickly removed from the skull, and sagittal hippocampal slices (400 μm thick) were cut on a vibratome (Leica). The slices were incubated at 35°C for 15 min in an incubation solution containing 119 mM NaCl, 2.5 mM KCl, 1 mM NaH2PO_4_, 26.2 NaHCO_3_, 11 mM glucose, 2 mM Na pyruvate, 1 mM Na ascorbate, 3 mM MgSO_4_, and 1.5 mM CaCl_2_. After incubation, the slices were transferred to aCSF solution at 23–24°C for 1 hour.

Field recordings were made with a concentric bipolar electrode positioned in the stratum radiatum of the CA1 region using an extracellular glass pipette (3–5 MΩ) filled with aCSF. Stimulation was delivered through a bipolar electrode (FHC, Bowdoin, ME, USA) placed in the SC-CA1 region. The SC circuit was visualized using differential interference contrast (DIC) microscopy at 4× magnification and identified by the ability to evoke short and constant latency fEPSPs at CA1 synapses by SC input stimulation. The test stimulation in all the fEPSP experiments was measured before the experiments (30–300 μA), and a test-pulse stimulation strength that evoked 50% of the maximum fEPSP was used.

To measure mEPSCs, the electrode was filled with an internal solution containing 135 mM Cs methane sulfonate, 8 mM NaCl, 10 mM HEPES, 0.5 mM EGTA, 4 mM Mg-ATP, 0.3 mM Na-GTP, and 5 mM QX-315 Cl; pH 7.25 with CsOH, 285 mOsm). Miniature currents were recorded in the presence of tetrodotoxin (1 μM TTX, Tocris) to block sodium currents and propagate action potentials.

**Stereotaxic FAM19A5 injection**

For stereotaxic injection of WT and MT FAM19A5(R58A, R59A), 3-month-old male and female mice were anesthetized with xylazine and ketamine. An injection cannula was stereotaxically inserted into the striatum (mediolateral, 2.0 mm from bregma; anteroposterior, 0.5 mm; dorsoventral, 3.5 mm) unilaterally (inserted into the right hemisphere). The infusion was performed at a rate of 0.2 μl/min, and 2 μl of FAM19A5 (diluted in PBS at a concentration of 5 μg/μl) or the same volume of PBS was injected into each mouse.

**Animal behavioral tests**

The behavioral tests were conducted by the NDIC, a contract research organization. The male mice used in all the behavioral tests were 9–11 months old. All the assays used littermates or age-matched animals. Behavioral tests were performed in a light- and noise-controlled behavioral room, where the animals were allowed to adapt for one hour before each behavioral test. All the behavioral data were analyzed in a blinded manner.

**Y-maze test**

The Y-shaped maze consisted of three identical arms (40 cm in length, 15 cm in height) at a 120° angle from each other. The mice were allowed to freely explore the three arms for 8 min with a luminosity of 40 lux. The sequence and the total number of arms entered were measured. Spontaneous alteration (%) was calculated as follows: the number of triads containing entries into all three arms/maximum possible alternations (the total number of arms entered – 2) × 100. Mice that constituted fewer than 10 total arm entries were omitted from the analysis.

**Morris water maze test**

The mice were trained to find the hidden platform (9 cm in diameter) in a stainless-steel pool (90 cm in diameter, 50 cm in height) filled with water (22 ± 1°C) to a depth of 30 cm. The platform was located 1 cm below the water level. In the acquisition phase, the mice were trained for six consecutive days with four trials/per day. Each trial ended either when an animal climbed onto the platform or when a maximum of 60 s had elapsed. Next, the mice were allowed to remain on the platform for 10 s. If they failed to locate the platform within 60 s, they were guided to the platform and left there for 10 s, and the escape time was recorded as 60 s. On day 7, for the probe test, the platform was removed, and the mice were allowed to explore freely for 60 s. The spatial memory ability of the individual animals in the probe test was determined by the following parameters: latency to target, number of target crossings, distance to the target, percentage in the SW quadrant, and percentage in the target. The target and quadrant SW refer to the location and quadrant where the hidden platform was located, respectively. These parameters were analyzed by SMART video tracking software (Panlab). Mice displaying abnormal behaviors, such as freezing and floating, were excluded from the final data.

**Extended references**

1. Kwak H, Cho EH, Cho EB, et al. Is FAM19A5 an adipokine? Peripheral FAM19A5 in wild-type, FAM19A5 knockout, and LacZ knockin mice. *Mol. Cells.* 2024:100125.

2. Mirdita M, von den Driesch L, Galiez C, Martin MJ, Söding J, Steinegger M. Uniclust databases of clustered and deeply annotated protein sequences and alignments. *Nucleic Acids Res.* 2017;45(D1):D170-D176.

3. Remmert M, Biegert A, Hauser A, Söding J. HHblits: lightning-fast iterative protein sequence searching by HMM-HMM alignment. *Nat Methods.* 2012;9(2):173-175.

4. Beaudoin GMJ, Lee SH, Singh D, et al. Culturing pyramidal neurons from the early postnatal mouse hippocampus and cortex. *Nat Protoc.* 2012;7(9):1741-1754.

5. Martin JA, Wang Z. Next-generation transcriptome assembly. *Nat Rev Genet.* 2011;12(10):671-682.

**Supplementary Figures**

**
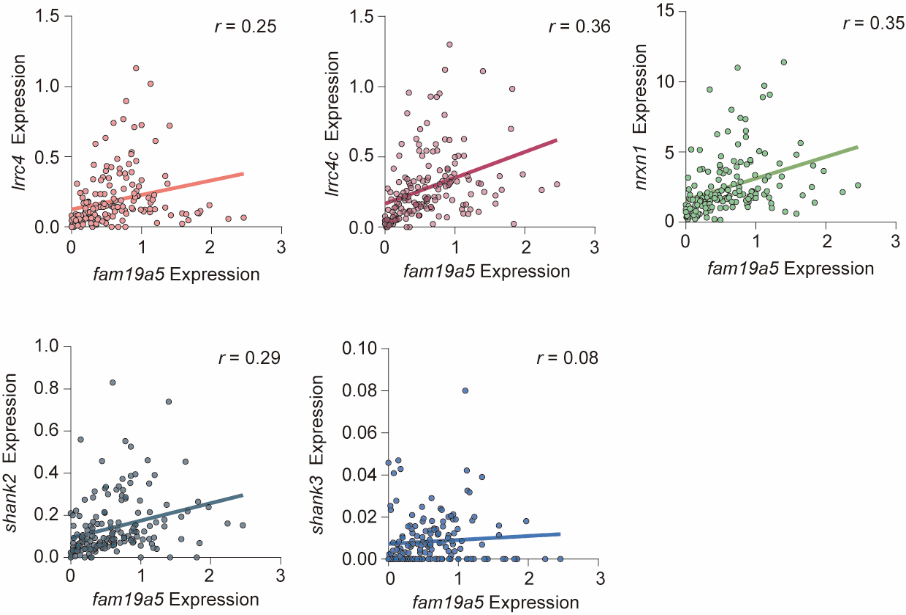
**

**Supplementary Figure 1** ***FAM19A5* transcript correlations with other synaptic adhesion molecules.** Correlations between the RNA transcript levels of *fam19a5* and synaptic adhesion molecules, *lrrc4*, *lrrc4c*, *nrxn1*, *shank2*, and *shank3* were examined across 168 neuron types. Correlation coefficient; *r*.

**
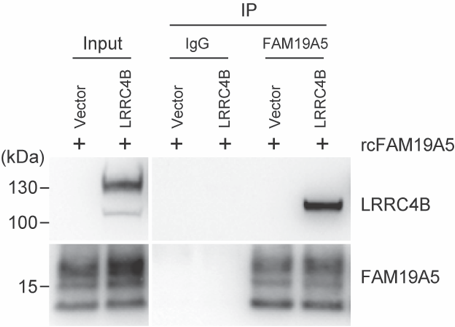
**

**Supplementary Figure 2** **FAM19A5 interacts with LRRC4B in HEK293 cell**. Treatment of LRRC4B-expressing HEK293 cells with recombinant (rc) FAM19A5 resulted in co-immunoprecipitation of the LRRC4B-FAM19A5 complex.

**~~
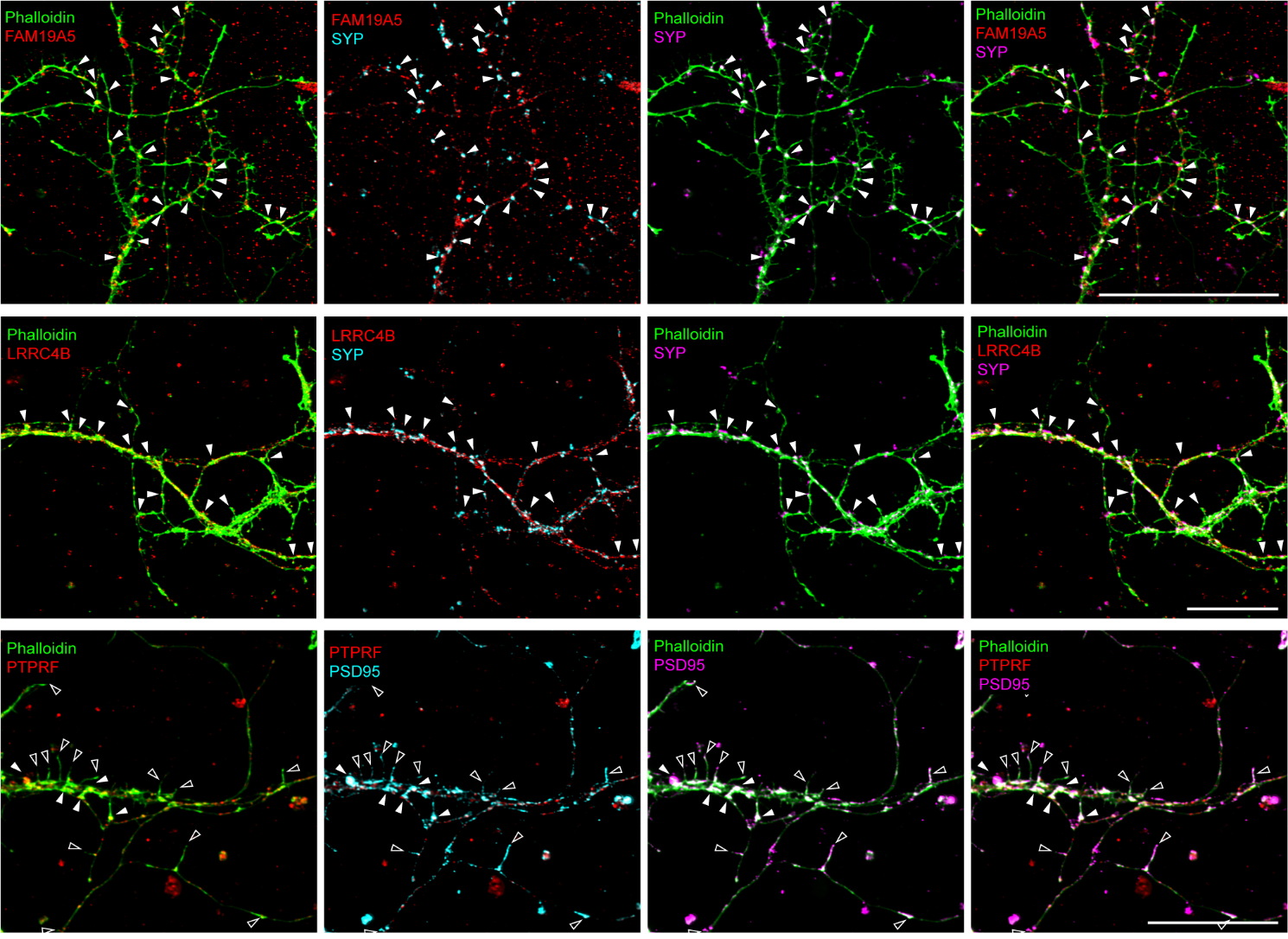
~~**

**Supplementary Figure 3 Immunofluorescence staining of neuronal dendrites showing the colocalization of synaptic proteins.** Images of 14 DIV hippocampal neurons stained with phalloidin (green). In the top row, neurons were costained with FAM19A5 (red) and synaptophysin (SYP, cyan or magenta). Solid arrowheads indicate the localization of FAM19A5 within dendritic spines and SYP at the presynaptic terminal. In the middle row, neurons were costained with LRRC4B (red) and SYP (cyan or magenta), showing the localization of LRRC4B on dendrites, while SYP localized to presynaptic terminals, marked by solid arrowheads. In the bottom row, neurons were costained with PTPRF (red) and PSD95 (cyan or magenta). Open arrowheads mark PTPRF-negative, PSD95-positive dendritic spines, while solid arrowheads indicate the localization of PTPRF at the presynaptic terminal and PSD95 at the postsynaptic spines. Scale bar: 20 μm.

**~~
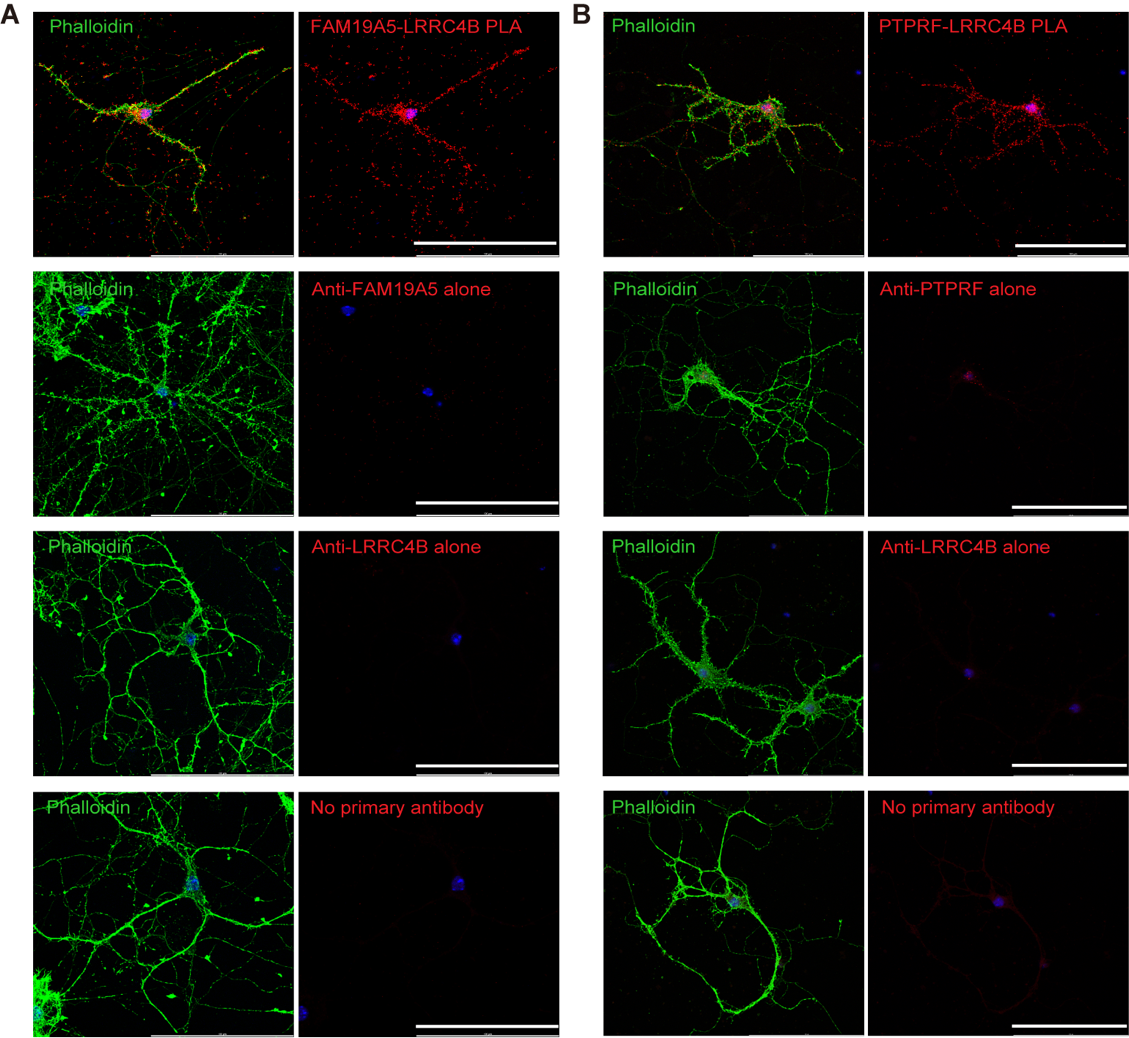
~~**

**Supplementary Figure 4 Proximity ligation assay (PLA) to detect interactions between FAM19A5, LRRC4B, and PTPRF in neuronal dendrites.** (A) The left two columns show the PLA results for FAM19A5 and LRRC4B. The first panel displays neurons stained with phalloidin (green) and PLA signal (red). The second panel shows only the PLA signal (red) for the FAM19A5-LRRC4B interaction with Hoechst (blue) staining the nucleus. The second row contains a negative control, with no PLA signal present when only anti-FAM19A5 antibody (C-A5-Ab) was used (middle panel). The third row shows another negative control with no LRRC4B primary antibody, demonstrating the absence of a PLA signal. The fourth row displays a control with no primary antibody, confirming that there is no PLA signal. (B) The right two columns show the PLA results for PTPRF and LRRC4B. In the top row, the first panel displays neurons stained with phalloidin (green). The second panel illustrates the PLA signal (red) for the PTPRF-LRRC4B interaction with Hoechst (blue) staining the nucleus. The second row contains a negative control using only the PTPRF primary antibody, with no interaction observed. The third row shows a control where only the anti-LRRC4B primary antibody was used, showing no PLA signal. Finally, the fourth row presents a control with no primary antibody, indicating no PLA signal. Scale bar: 100 µm.

**
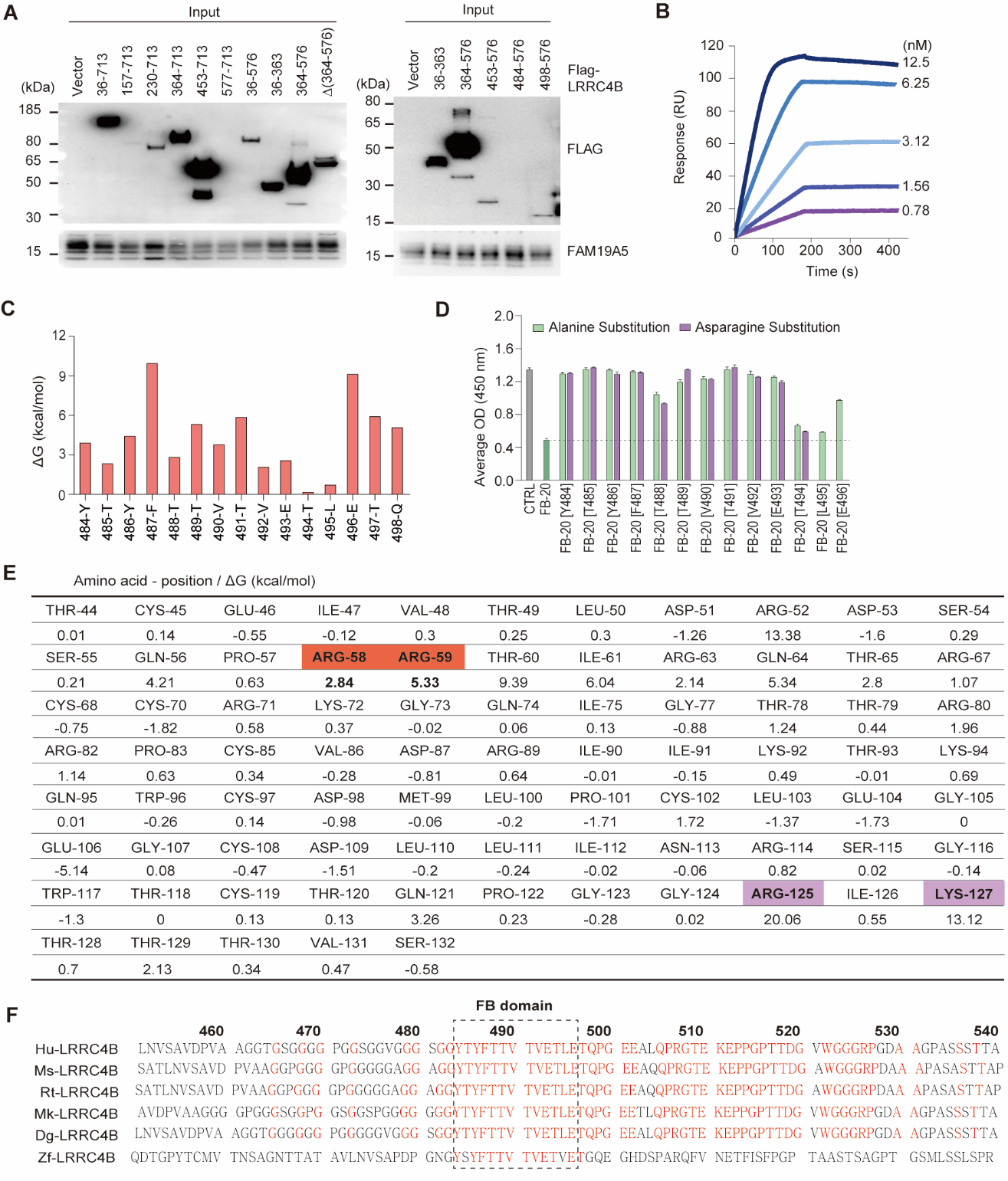
**

**Supplementary Figure 5 Identification of binding sites for the interaction between LRRC4B and FAM19A5.** (A) Western blot for input samples for Co-IP, as shown in Fig. 2B. (B) SPR measurement to determine the binding affinity between LRRC4B and FAM19A5. The signals were collected from a chip coated with FB-containing protein (LRRC4B(453-576)-hFc) while treating FAM19A5 in a dose-dependent manner (0.78~12.5 nM, K_a_ = 4.892 × 10^6^ M^-1^ s^-1^, K_d_ = 1.570 × 10^-4^ s^-1^, K_D_ = 3.209 × 10^-11^ M). (C) Predicted change in binding free energy between FAM19A5 and the LRRC4B FB region upon Ala substitution at each residue. (D) The key residues for FAM19A5-LRRC4B were confirmed via ELISA using the mutated FB proteins (captured by LRRC4B(36-576)-hFc, detected by HRP-conjugated C-A5-Ab). (E) Predicted change in the binding free energy of FAM19A5 after Ala scanning. Competitive binding sites for the FB domain and NS101 (orange) and key residues for binding (pink) are highlighted. (F) Sequence alignment of the LRRC4B FB sequences across species, including humans.

**
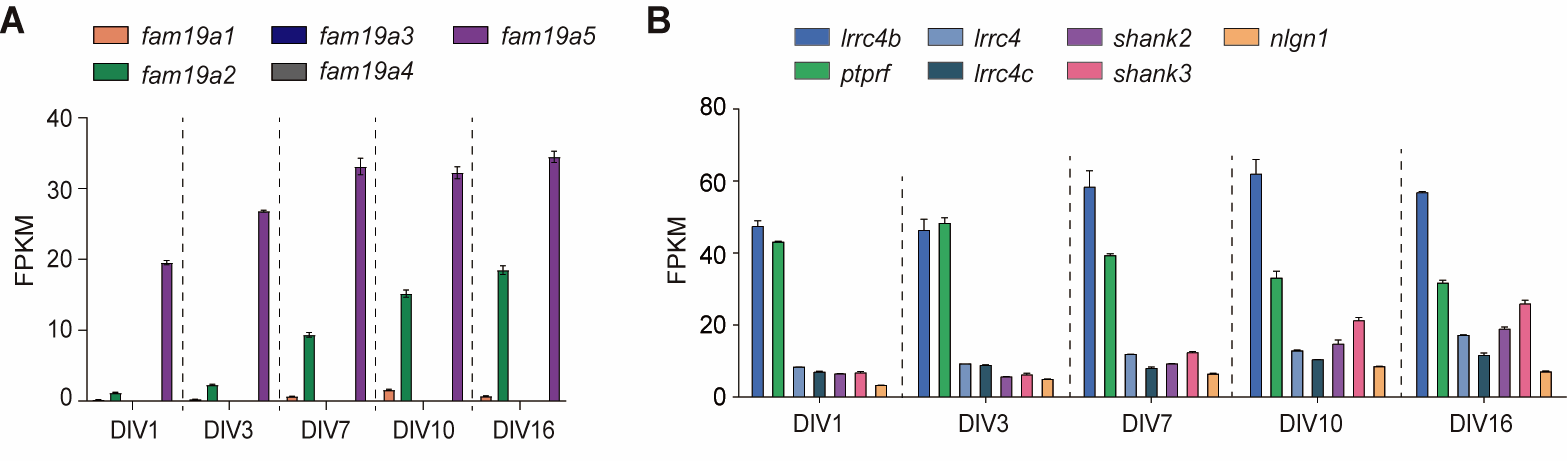
**

**Supplementary Figure 6 RNA expression levels of the FAM19A family and synapse adhesion molecules**. RNA transcription levels of (A) the fam19a family and (B) synapse adhesion molecules in primary hippocampal neurons at the indicated time points (n = 3 per group). The data are presented as the mean ± SEM.

**
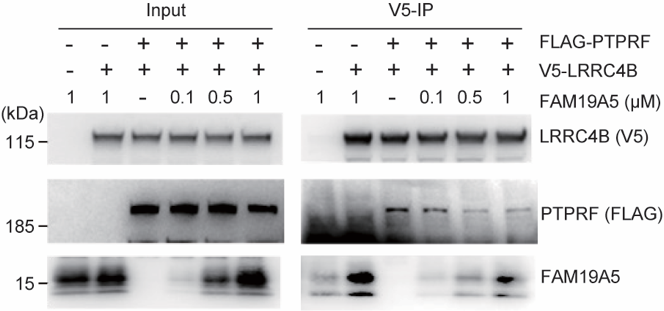
**

**Supplementary Figure 7 FAM19A5 binding to LRRC4B dissociates the LRRC4B-PTPRF complex.** Co-IP experiments demonstrated an interaction between LRRC4B and PTPRF. This interaction was inhibited by FAM19A5 treatment in a dose-dependent manner.

**
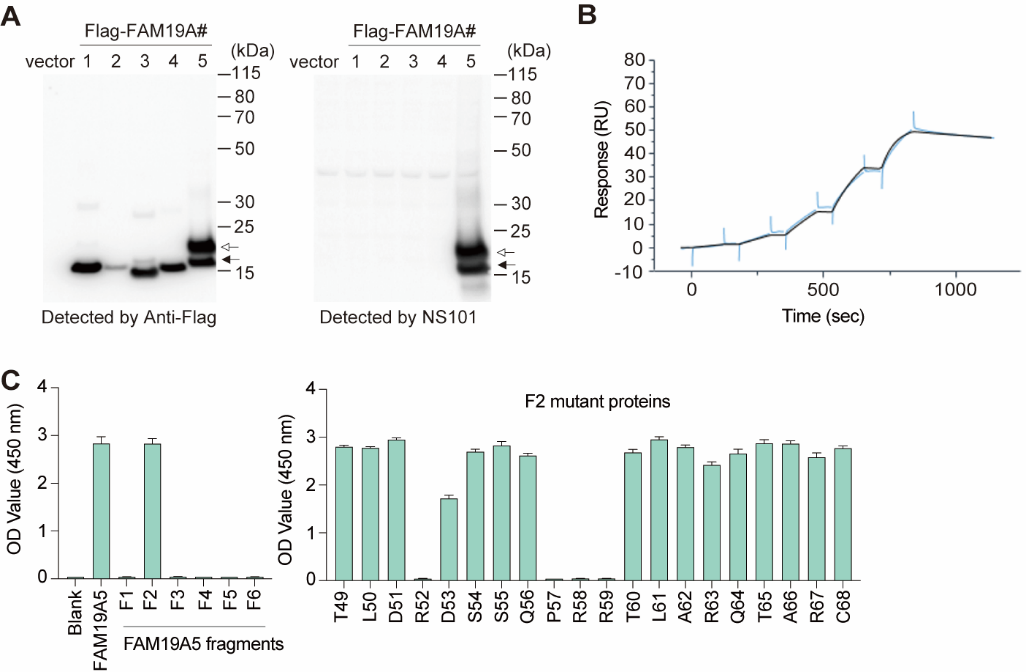
**

**Supplementary Figure 8 Characterization of NS101.** (A) Western blot analysis of FAM19A family members (FAM19A#) detected by an anti-Flag antibody (left) and NS101 (right). Open arrow: glycosylated FAM19A5; solid arrow: nonglycosylated FAM19A5. (B) SPR measurement to determine the binding affinity of NS101 for FAM19A5. The signals were collected from a chip coated with FAM19A5 and treated with increasing concentrations of NS101 (0.12~10 nM, K_a_ = 2.11 × 10^6^ M^-1^ s^-1^, K_d_ = 2.34 × 10^-4^ s^-1^, K_D_ = 1.11 × 10^-10^ M). (C) Epitope mapping of FAM19A5. The binding of NS101 to FAM19A5 and synthetic peptides (F1~F6 and F2 mutant peptides, see Supplementary Table 3 and 4) was evaluated via ELISA (captured by the FAM19A5 fragment bound by NS101 and detected by HRP-conjugated hIgG). The amino acid residues are numbered according to the human FAM19A5 sequence.

**
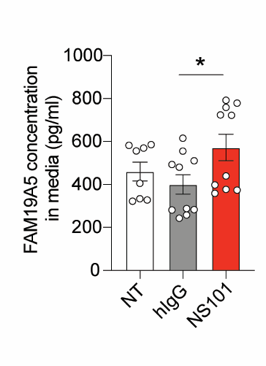
**

**Supplementary Figure 9 FAM19A5 inhibition by NS101 in cultured neuron.** FAM19A5 levels in the media secreted from 11 DIV primary hippocampal neurons after antibodies treatment were measured (10 nM for 48 hours, n = 8-10). The data are presented as the mean ± SEM. One-way ANOVA with Bonferroni’s multiple comparisons test was used to calculate P values (F_2, 25_ = 2.987, P = 0.0687). *, P = 0.047

**
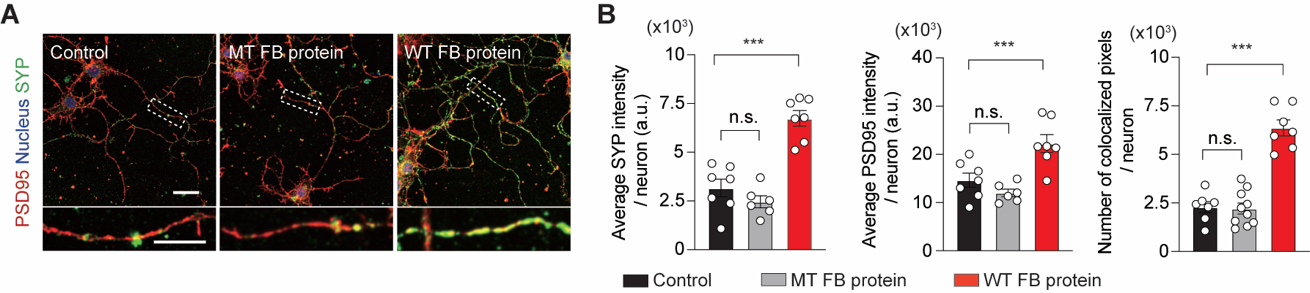
**

**Supplementary Figure 10 Inhibition of FAM19A5 via the FB protein and its effect on synapse**. (A) Representative image of cultured hippocampal neurons treated with WT LRRC4B (453–576-hFc) or MT LRRC4B (453–576, T488A, T489A)-hFc FB-containing proteins stained with SYP (green) and PSD95 (red). Nuclei (blue). Scale bar, 20 μm, 10 μm (enlarged). (B) Quantification of the intensity and colocalization of the markers shown in (n = 6 to 7 per group). The data are presented as the mean ± SEM. One-way ANOVA with Tukey’s multiple comparisons test was used to calculate P values (SYP intensity: F_2, 17_ = 33.06, P < 0.0001; PSD95 intensity: F_2, 17_ = 12.72, P < 0.001; number of colocalized pixels: F_2, 21_ = 50.47, P < 0.0001). n.s., not significant; ***, P < 0.001.

**
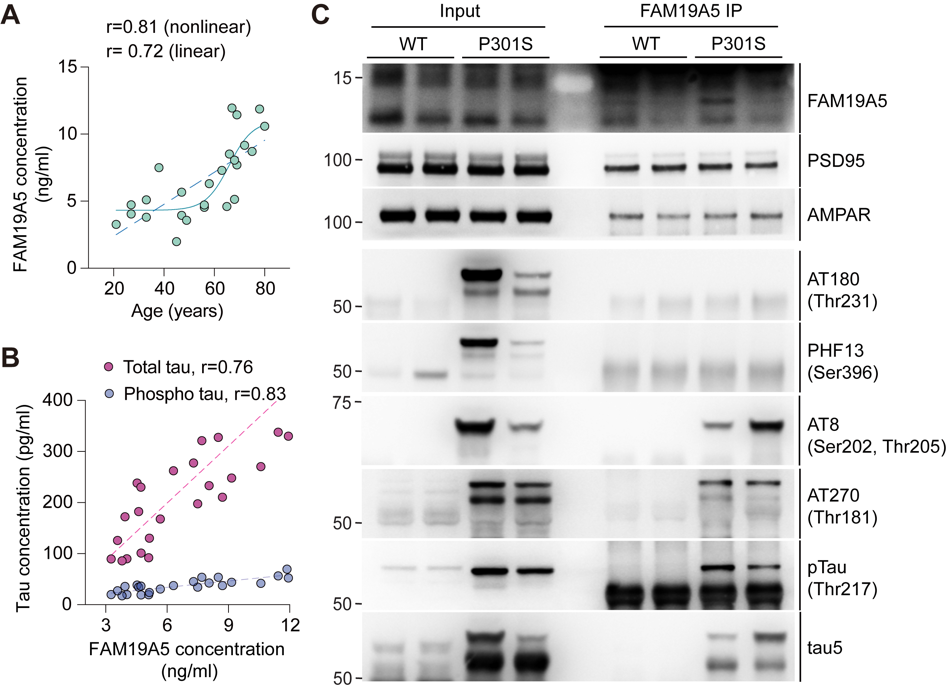
**

**Supplementary Figure 11 Interaction between FAM19A5 and phosphorylated tau proteins in the brains of P301S mice.** (A) Correlation between FAM19A5 and age in human CSF. (B) Correlations between tau and FAM19A5 in human CSF. Correlation coefficient; r. (C) Co-IP using the synaptosome fraction of P301S mouse brains to investigate the interaction between FAM19A5, synaptic molecules and phosphorylated Tau proteins.


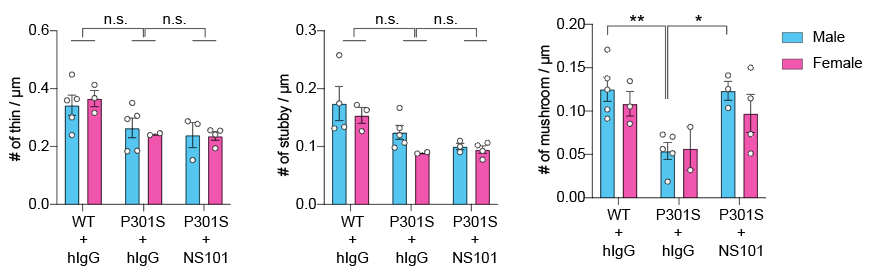


Supplementary Figure 12. Comparison of NS101-mediated restoration effects on dendritic spine subtypes by sex. Dendritic spines were classified into thin, stubby, and mushroom types and analyzed separately in male and female mice. Statistical analysis was conducted using two-way ANOVA (Drug effect: F_1, 16_ = 0.9592, P = 0.3420; sex effect: F_2, 16_ = 7.625, P = 0.0047) followed by Šídák's multiple comparisons test (thin: F_2, 16_ = 6.868, P < 0.01; stubby: F_2, 15_ = 8.686, P < 0.01; mushroom: F_2, 16_ = 7.625, P < 0.005). For thin spines, the P values were as follows: male WT + hIgG vs. P301S + hIgG, 0.1619; male P301S + hIgG vs. P301S + NS101, 0.8609; female WT + hIgG vs. P301S + hIgG, 0.1231; and female P301S + hIgG vs. P301S + NS101, 0.9921. For stubby spines: male WT + hIgG vs. P301S + hIgG, 0.0816; male P301S + hIgG vs. P301S + NS101, 0.5560; female WT + hIgG vs. P301S + hIgG, 0.0987; and female P301S + hIgG vs. P301S + NS101, 0.9790. For mushroom spines: male WT + hIgG vs. P301S + hIgG, 0.0056; male P301S + hIgG vs. P301S + NS101, 0.0193; female WT + hIgG vs. P301S + hIgG, 0.2244; and female P301S + hIgG vs. P301S + NS101, 0.3722. Data are presented as mean ± SEM.

**
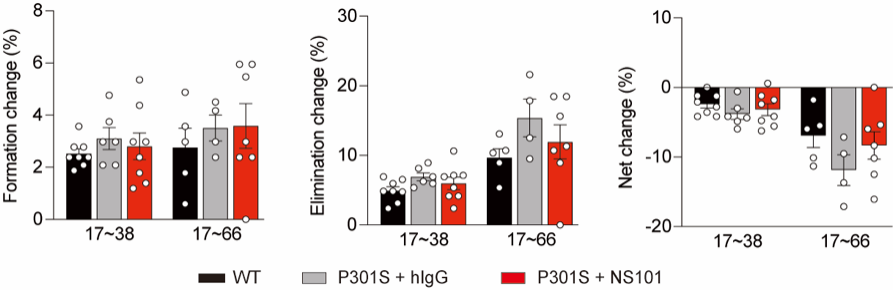
**

**Supplementary Figure 13 Quantification of spine dynamics at days 17~38 and 17~66 in P301S mice.** The formation and elimination rates and their net changes after the intraperitoneal administration of 30 mg/kg NS101 (n = 4 to 8 per group). The data are presented as the mean ± SEM.

**
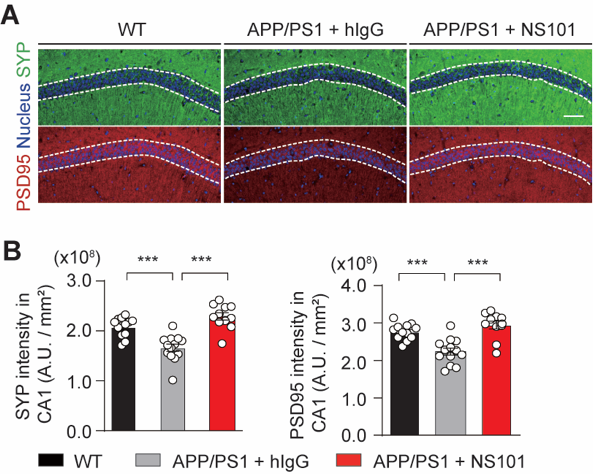
**

**Supplementary Figure 14 Restoration of synaptic marker expression in the brains of APP/PS1 mice after NS101 treatment.** (A) Representative image of the hippocampal CA1 region in APP/PS1 mice after IV administration of NS101 or hIgG (30 mg/kg each) stained with SYP (green) and PSD95 (red). Scale bar, 100 μm. (B) Quantified intensity of synaptic marker proteins from (A) (n = 11-12 per group). The data are presented as the mean ± SEM. One-way ANOVA with Tukey’s multiple comparisons test in (B) was used to calculate P values (SYP intensity: F_2, 33_ = 22.11, P < 0.0001; PSD95 intensity: F_2, 33_ = 17.77, P < 0.0001). ***, P < 0.001.

**
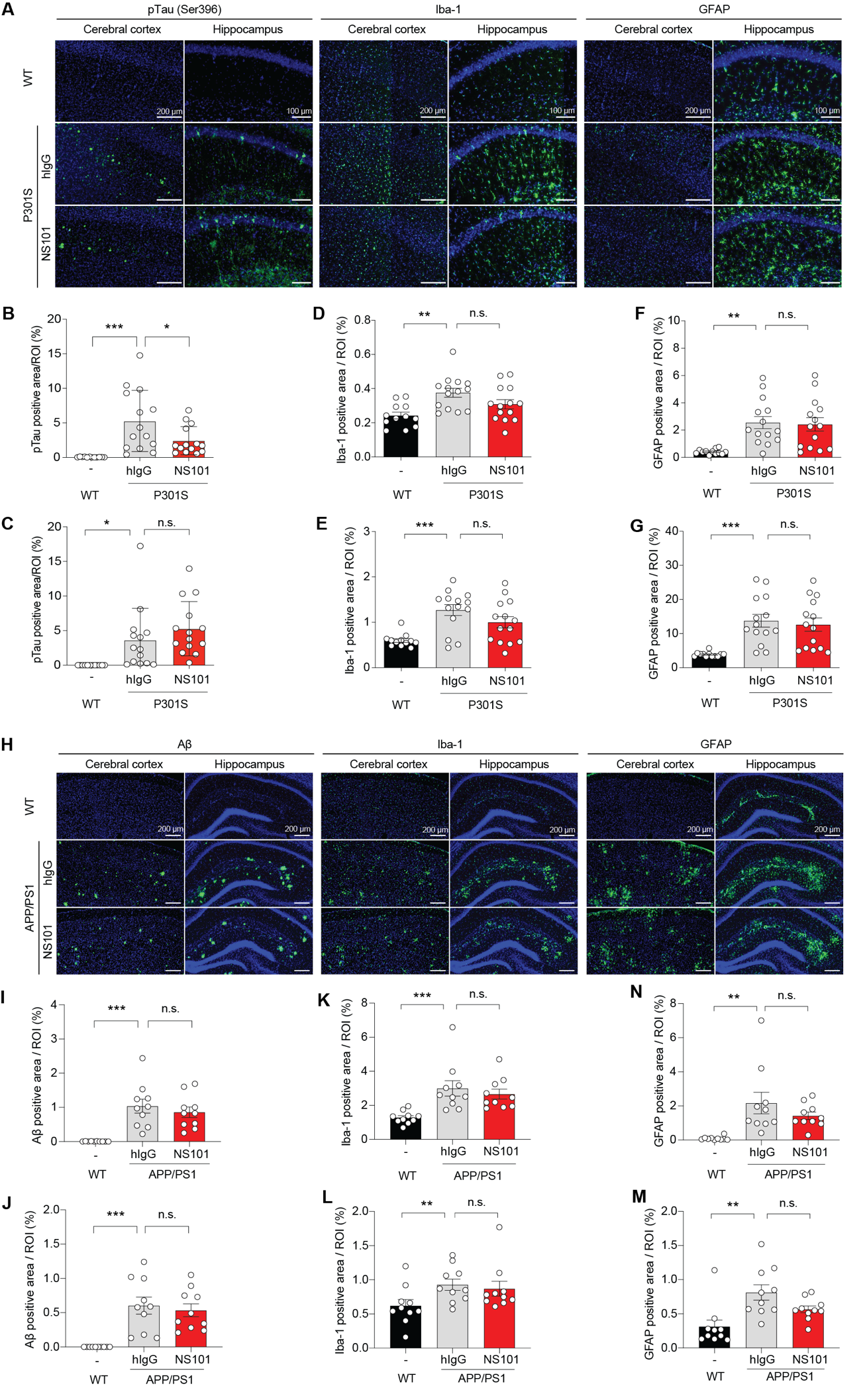
**

**Supplementary Figure 15** **Effect of NS101 on tau phosphorylation, amyloid plaque formation, and glial activation.** Eight-month-old APP/PS1 mice and 9-month-old P301S mice received 10 mg/kg NS101 weekly for eight weeks, followed by immunostaining of the aggregates and glial cells. Phosphorylated tau at serine 396 positive area in the (A) cortex and (B) hippocampus of P301S mice was analyzed. The quantification of amyloid plaques was performed in the (C) cortex and (D) hippocampus of APP/PS1 mice. Microglia-positive areas in the (E) cortex and (F) hippocampus of P301S mice were quantified. The microglia-positive areas in the (G) cortex and (H) hippocampus of APP/PS1 mice were further analyzed. The astrocyte-positive areas in the (I) cortex and (J) hippocampus of P301S mice were analyzed. Additionally, the astrocyte-positive area in the (K) cortex and (L) hippocampus of APP/PS1 mice were analyzed. The data are presented as the mean ± SEM. n=10-14 per group, One-way ANOVA followed by Tukey’s multiple comparison test was used to calculate P values (B: F_2, 37_ = 10.68, P < 0.001; C: F_2, 37_ = 7.269, P < 0.001; D: F_2, 37_ = 6.894, P < 0.05; E: F_2, 37_ = 9.336, P < 0.001; F: F_2, 37_ = 8.309, P = 0.001; G: F_2, 37_ = 10.20, P < 0.001; I: F2, 27 = 6.499, P = 0.005; J: F_2, 27_ = 13.47, P < 0.0001; K: F_2, 27_ = 8.193, P < 0.005; N: F_2, 27_ = 7.411, P < 0.01; M: F_2, 27_ = 7.652, P < 0.005). Two-tailed unpaired t-test (for L) was used to calculate the P value (WT vs. APP/PS1+hIgG: t_18_ = 2.534, P < 0.05; APP/PS1+hIgG vs. APP/PS1+NS101: t_18_ = 0.4323, P = 0.6706). n.s., not significant; *, P = 0.0296, ***, P < 0.001(A); ***, P < 0.001 (B); ***, P < 0.001 (C); ***, P < 0.001 (D); **, P = 0.002 (E); ***, P < 0.001 (F); ***, P < 0.001 (G); *, P = 0.0078 (H); **, P = 0.0019 (I); ***, P < 0.001 (J); **, P = 0.0018 (K); **, P = 0.0017 (L).

**Supplementary Table 1.** Primers for cloning.

| **Insert** | **Primer Sequence** | |
| --- | --- | --- |
| LRRC4B 36-713 | For | GATGACGACAAGCTTGCCGGTGGAGGTGGAG |
|  | Rev | GTTTTTGTTCGGATCCTCAGATCTGCGTCTCTTGC |
| LRRC4B 157-713 | For | GATGACGACAAGCTTCTGTCCAAGCTGCGGG |
|  | Rev | GTTTTTGTTCGGATCCTCAGATCTGCGTCTCTTGC |
| LRRC4B 230-713 | For | GATGACGACAAGCTTCGCCTGGAGGAGCTGG |
|  | Rev | GTTTTTGTTCGGATCCTCAGATCTGCGTCTCTTGC |
| LRRC4B 364-713 | For | GATGACGACAAGCTTCCCGTCATCGTGGAGC |
|  | Rev | GTTTTTGTTCGGATCCTCAGATCTGCGTCTCTTGC |
| LRRC4B 453-713 | For | GATGACGACAAGCTTGTCTCGGCCGTGGACC |
|  | Rev | GTTTTTGTTCGGATCCTCAGATCTGCGTCTCTTGC |
| LRRC4B 577-713 | For | GATGACGACAAGCTTATCATCATCGGCTGCTTC |
|  | Rev | GTTTTTGTTCGGATCCTCAGATCTGCGTCTCTTGC |
| LRRC4B 36-576 | For | GATGACGACAAGCTTGCCGGTGGAGGTGGAG |
|  | Rev | GTTTTTGTTCGGATCCTCATTTGGTGGTCTTCATG |
| LRRC4B 36-363 | For | GATGACGACAAGCTTGCCGGTGGAGGTGGAG |
|  | Rev | GTTTTTGTTCGGATCCTCACGCATAGCAGGTG |
| LRRC4B 364-576 | For | GATGACGACAAGCTTCCCGTCATCGTGGAGC |
|  | Rev | GTTTTTGTTCGGATCCTCATTTGGTGGTCTTCATG |
| LRRC4B D364-576 | For | GATGACGACAAGCTTGCCGGTGGAGGTGGAG |
|  | InRev | GAAGCAGCCGATGATGATCGCATAGCAGGTGAAATG |
|  | InFor | CATTTCACCTGCTATGCGATCATCATCGGCTGCTTC |
|  | Rev | GTTTTTGTTCGGATCCTCAGATCTGCGTCTCTTGC |
| LRRC4B 453-576 | For | GATGACGACAAGCTTGTCTCGGCCGTGGACC |
|  | Rev | GTTTTTGTTCGGATCCTCATTTGGTGGTCTTCATG |
| LRRC4B 484-576 | For | GATGACGACAAGCTTTACACCTACTTCACCACGGTG |
|  | Rev | GTTTTTGTTCGGATCCTCATTTGGTGGTCTTCATG |
| LRRC4B 498-576 | For | GATGACGACAAGCTTCAGCCCGGAGAGGAGG |
|  | Rev | GTTTTTGTTCGGATCCTCATTTGGTGGTCTTCATG |
| LRRC4C 354-527 | For | GATGACGACAAGCTTCCGGTGATTGTGGAGCC |
|  | Rev | GTTTTTGTTCGGATCCTCATTTGGTAGTCTTCATGACC |
| LRRC4 353-527 | For | GATGACGACAAGCTTCCCTTCATCATGGACGC |
|  | Rev | GTTTTTGTTCGGATCCTCACTTGGTGGTCTTCATGAC |
| LRRC4B 453-576  (T488A, T489A) | For | GATGACGACAAGCTTGTCTCGGCCGTGGACC |
|  | InRev | CACGGTCACGGCCGCGAAGTAGGTGTAG |
|  | InFor | CTTCGCGGCCGTGACCGTGGAGAC |
|  | Rev | GTTTTTGTTCGGATCCTCATTTGGTGGTCTTCATG |
| LRRC4B 453-576  (T488S, T489S) | For | GATGACGACAAGCTTGTCTCGGCCGTGGACC |
|  | InRev1 | GTCTCCACGGTCACAGACGAGAAGTAGGTGTAG |
|  | InFor1 | CTACACCTACTTCTCGTCTGTGACCGTGGAGAC |
|  | InRev2 | CAGACACCGTCAGACGAGGGCCCTG |
|  | InFor2 | CAGGGCCCTCGTCTGACGGTGTCTG |
|  | Rev | GTTTTTGTTCGGATCCTCATTTGGTGGTCTTCATG |
| LRRC4B 36-713  (T488A, T489A) | For | GATGACGACAAGCTTGCCGGTGGAGGTGGAG |
|  | InRev | CACGGTCACGGCCGCGAAGTAGGTGTAG |
|  | InFor | CTACACCTACTTCGCGGCCGTGACCGTG |
|  | Rev | GTTTTTGTTCGGATCCTCAGATCTGCGTCTCTTGC |
| FAM19A5 (R58A, R59A) | For | CAGCCAGCCTGCCGCCACGATC |
|  | InRev | GATCGTGGCGGCAGGCTGGCTG |
|  | InFor | ATGGAGACAGACACACTCCTGCTATGGGTACTG |
|  | Rev | CAGTACCCATAGCAGGAGTGTGTCTGTCTCCAT |
| FAM19A5 (R125A, K127A) | For | CGGGGCCATAGCCACCACCACGGTCTC |
|  | InRev | GAACGAAATAGACAGATCGCTGAGATAGGTGCCTCAC |
|  | InFor | GTGAGGCACCTATCTCAGCGATCTGTCTATTTCGTTC |
|  | Rev | GAGACCGTGGTGGTGGCTATGGCCCCG |
| PTPRF (30-1263) | For | GTTCCACTGGTGACAAGCTTGACAGCAAACCTGTC |
|  | Rev | CCACCGCCGAATTCCCACAGCATCTCCGGC |

**Supplementary Table 2.** The sequences of point mutated FB-20 proteins

| **Name** | **Manufacturer** | **Lot No.** | **Amino acid sequence** |
| --- | --- | --- | --- |
| FB-20 | Anygen | K210024 | GYTYFTTVTVETLETQPGEE |
| FB-20[Y484A] | Anygen | K210410 | G**A**TYFTTVTVETLETQPGEE |
| FB-20[Y485A] | Anygen | K210411 | GY**A**YFTTVTVETLETQPGEE |
| FB-20[Y486A] | Anygen | K210412 | GYT**A**FTTVTVETLETQPGEE |
| FB-20[Y487A] | Anygen | K210413 | GYTY**A**TTVTVETLETQPGEE |
| FB-20[Y488A] | Anygen | K210414 | GYTYF**A**TVTVETLETQPGEE |
| FB-20[Y489A] | Anygen | K210415 | GYTYFT**A**VTVETLETQPGEE |
| FB-20[Y490A] | Anygen | K210416 | GYTYFTT**A**TVETLETQPGEE |
| FB-20[Y491A] | Anygen | K210417 | GYTYFTTV**A**VETLETQPGEE |
| FB-20[Y492A] | Anygen | K210418 | GYTYFTTVT**A**ETLETQPGEE |
| FB-20[Y493A] | Anygen | K210419 | GYTYFTTVTV**A**TLETQPGEE |
| FB-20[Y494A] | Anygen | K210420 | GYTYFTTVTVE**A**LETQPGEE |
| FB-20[Y495A] | Anygen | K210421 | GYTYFTTVTVET**A**ETQPGEE |
| FB-20[Y496A] | Anygen | K210422 | GYTYFTTVTVETL**A**TQPGEE |
| FB-20[Y484N] | Anygen | K210423 | G**N**TYFTTVTVETLETQPGEE |
| FB-20[Y485N] | Anygen | K210424 | GY**N**YFTTVTVETLETQPGEE |
| FB-20[Y486N] | Anygen | K210425 | GYT**N**FTTVTVETLETQPGEE |
| FB-20[Y487N] | Anygen | K210426 | GYTY**N**TTVTVETLETQPGEE |
| FB-20[Y488N] | Anygen | K210427 | GYTYF**N**TVTVETLETQPGEE |
| FB-20[Y489N] | Anygen | K210428 | GYTYFT**N**VTVETLETQPGEE |
| FB-20[Y490N] | Anygen | K210429 | GYTYFTT**N**TVETLETQPGEE |
| FB-20[Y491N] | Anygen | K210430 | GYTYFTTV**N**VETLETQPGEE |
| FB-20[Y492N] | Anygen | K210431 | GYTYFTTVT**N**ETLETQPGEE |
| FB-20[Y493N] | Anygen | K210432 | GYTYFTTVTV**N**TLETQPGEE |
| FB-20[Y494N] | Anygen | K210433 | GYTYFTTVTVE**N**LETQPGEE |

**Supplementary Table 3.** The sequences of the FAM19A5 fragment proteins

| **Name** | **Manufacturer** | **Lot No.** | **Amino acid sequence (C → S)** |
| --- | --- | --- | --- |
| F1 | Peptron | 15-103901 | QFLKEGQLAAGTSEIVTLDR-GGGSC-BSA |
| F2 | Peptron | 15-103902 | TLDRDSSQPRRTIARQTARS-GGGSC-BSA |
| F3 | Peptron | 15-103903 | TARSASRKGQIAGTTRARPA-GGGSC-BSA |
| F4 | Peptron | 15-103904 | ARPASVDARIIKTKQWSDML-GGGSC-BSA |
| F5 | Peptron | 15-103905 | SDMLPSLEGEGSDLLINRSG-GGGSC-BSA |
| F6 | Peptron | 15-103906 | NRSGWTSTQPGGRIKTTTVS-GGGSC-BSA |

**Supplementary Table 4.** The sequences of point mutated F2 fragments of FAM19A5

| **Name** | **Manufacturer** | **Lot No.** | **Amino acid sequence** |
| --- | --- | --- | --- |
| F2-01 | Peptron | 16-105901 | TADRDSSQPRRTIARQTARC-BSA |
| F2-02 | Peptron | 16-105902 | TLARDSSQPRRTIARQTARC-BSA |
| F2-03 | Peptron | 16-105903 | TLDRASSQPRRTIARQTARC-BSA |
| F2-04 | Peptron | 16-105904 | TLDRDASQPRRTIARQTARC-BSA |
| F2-05 | Peptron | 16-105905 | TLDRDSAQPRRTIARQTARC-BSA |
| F2-06 | Peptron | 16-105906 | TLDRDSSAPRRTIARQTARC-BSA |
| F2-07 | Peptron | 16-105907 | TLDRDSSQARRTIARQTARC-BSA |
| F2-08 | Peptron | 16-105908 | TLDRDSSQPRATIARQTARC-BSA |
| F2-09 | Peptron | 16-105909 | TLDRDSSQPRRAIARQTARC-BSA |
| F2-10 | Peptron | 16-105910 | TLDRDSSQPRRTAARQTARC-BSA |
| F2-11 | Peptron | 16-105911 | TLDRDSSQPRRTIRRQTARC-BSA |
| F2-12 | Peptron | 16-105912 | TLDRDSSQPRRTIAAQTARC-BSA |
| F2-13 | Peptron | 16-105913 | TLDRDSSQPRRTIARQTVRC-BSA |
| F2-14 | Peptron | 17-99901 | ALDRDSSQPRRTIARQTARC-BSA |
| F2-15 | Peptron | 17-99902 | TLDADSSQPRRTIARQTARC-BSA |
| F2-16 | Peptron | 17-99903 | TLDRDSSQPARTIARQTARC-BSA |
| F2-17 | Peptron | 17-99904 | TLDRDSSQPRRTIARATARC-BSA |
| F2-18 | Peptron | 17-99905 | TLDRDSSQPRRTIARQAARC-BSA |
| F2-19 | Peptron | 17-99906 | TLDRDSSQPRRTIARQTAAC-BSA |
| F2-20 | Peptron | 17-99907 | TLDRDSSQPRRTIARQTARA-GGGSC-BSA |

**Supplementary Table 5.** The antibodies used in this study.

| Mouse monoclonal anti-Synaptophysin | Sigma-Aldrich | Cat# S5768; RRID: AB_477523 |
| --- | --- | --- |
| Mouse monoclonal anti-PSD95 | Invitrogen | Cat# MA1-045; RRID: AB_325399 |
| Rabbit polyclonal anti-PSD95 | Invitrogen | Cat# 51-6900; RRID: AB_87705 |
| Mouse monoclonal anti-Tau5 | Invitrogen | Cat# AHB0042; RRID: AB_1502093 |
| Rabbit polyclonal anti-phospho ser396 Tau | Genetex | Cat# GTX50166; RRID: AB_11176496 |
| Mouse monoclonal anti-beta amyloid | Bio Legend | Cat# 803015; RRID: AB_2565328 |
| Rabbit polyclonal anti-iba1 | wako | Cat# 019-19741; RRID: AB_839504 |
| Rat monoclonal anti-GFAP | Invitrogen | Cat# 13-0300; RRID: AB_86543 |
| Mouse monoclonal anti-NGL-3/LRRC4B | R&D Systems | Cat# MAB69191; RRID: AB_11129846 |
| Rabbit polyclonal anti-NGL-3/LRRC4B | Alomone labs | Cat# ANR-163; RRID: AB_2827334 |
| Mouse monoclonal anti-PTPRF | Invitrogen | Cat# MA5-27668; RRID: AB_2735337 |
| Rabbit polyclonal anti-PTPRF | Sigma-Aldrich | Cat# SAB4200321; RRID: AB_10896235 |
| Goat polyclonal anti-PTPRF | R&D Systems | Cat# AF3004; RRID: AB_2174681 |
| Mouse monoclonal anti-FLAG M2 | Sigma-Aldrich | Cat# F1804; RRID: AB_262044 |
| Mouse monoclonal anti-V5 | Invitrogen | Cat# R960CUS; RRID: AB_2792973 |
| Donkey anti-Human IgG-HRP | Jackson ImmunoResearch | Cat# 715-035-149; RRID: AB_2340495 |
| Donkey anti-Mouse IgG-HRP | Jackson ImmunoResearch | Cat# 715-035-150; RRID: AB_2340770 |
| Donkey anti-Mouse IgG | abcam | Cat# ab96878; RRID: AB_10680869 |
| Donkey anti-Human IgG-Cy3 | Jackson ImmunoResearch | Cat# 709-165-149; RRID: AB_2340535 |
| Donkey anti-Rabbit IgG-Alexa 647 | Invitrogen | Cat# A31573; RRID: AB_2536183 |
| Donkey anti-Goat IgG-Alexa 555 | Invitrogen | Cat# A21432; RRID: AB_141788 |
| Rabbit anti-Human IgG Heavy chain | Invitrogen | Cat# SA5-10223; RRID: AB_2665319 |
| Rabbit anti-Human IgG Fc-HRP | Invitrogen | Cat# 31423; RRID: AB_228409 |
| Goat anti-Human kappa light chain-HRP | Invitrogen | Cat# A18853; RRID: AB_2535630 |
| Goat anti-mouse IgG-cy3 | Invitrogen | Cat# A10521; RRID: AB_1500665 |
| anti-Human isotype control | Invitrogen | Cat# 31154; RRID: AB_243591 |
| Anti-Human IgG1, kappa isotype control | Sino Biological | Cat# HG1K; RRID: AB_3661945 |
